# Effectiveness of an online application of the health action process approach (HAPA) theory on oral hygiene intervention in young adults with fixed orthodontic appliances: a randomized controlled trial

**DOI:** 10.1186/s12903-022-02219-w

**Published:** 2022-05-19

**Authors:** Weizi Wu, Lulu Hu, Yihan Chen, Feiran Cao, Sixie Ding, Tingting Wu, Jianguang Xu

**Affiliations:** 1 Department of Stomatology, Anhui Medical University, Hefei, China; 2 Department of Orthodontics, Affiliated Hospital of Stomatology, Anhui Medical University Hefei, 69 Meishan Road, Hefei, Anhui Province China

**Keywords:** Oral hygiene, Behavioral intervention, Compliance, Oral health behavior, Orthodontics, Mini-program

## Abstract

**Background:**

This randomized controlled trial aimed to evaluate the effectiveness of an online application based on HAPA theory on oral hygiene promotion in young adults with fixed orthodontic appliances.

**Methods:**

A WeChat mini-program (called “Clean Teeth”) based on HAPA theory was designed beforehand to improve oral-health behaviors and oral hygiene, and 44 participants aged 17–29 with fixed orthodontic appliances were recruited. Participants of the experimental group (n = 22) received the “Clean Teeth” mini-program, in addition to care as usual, and the control group (n = 22) only received routine oral health education. Data were collected during three orthodontic check-ups: baseline (T0), 6 weeks of follow-up (T1), and 12 weeks of follow-up (T2). All participants completed questionnaires assessing oral health behaviors and the psychosocial factors of the HAPA model and accepted the clinical examinations involving the dental plaque index and the gingival bleeding index.

**Results:**

After a 12-week intervention, the plaque index and gingival bleeding index in the experimental group were significantly lower than that in the control group. The psycho-social parameters of social effects, expected outcomes, and action control were improved significantly after treatment, among which social effects increased significantly only in the experimental group but not in the control group.

**Conclusions:**

The HAPA theory-based mini-program had positive effects on oral-health behavior promotion and oral hygiene among young adults with fixed orthodontic appliances.

*Trial registration* This study was retrospectively registered in the Chinese Clinical Trial Registry, with the number CTR2200056731, dated 12/02/2022. http://www.chictr.org.cn/index.aspx.

**Supplementary Information:**

The online version contains supplementary material available at 10.1186/s12903-022-02219-w.

## Background

Fixed orthodontic appliances with increasing accuracy and reliability are used in daily clinical practice. However, how to effectively avoid enamel demineralization and maintain good levels of oral hygiene during treatment is still a problem to be overcome [1, 2].

Oral hygiene education, mostly involving verbal instructions and model demonstrations, is the most common method used by orthodontists in clinical practice [3]. However, it is difficult for patients to understand and is often easily forgotten. Studies have pointed out that personalized oral hygiene education can reduce plaque attachment and improve patient compliance for plaque control [4, 5].

**Fig. 1:**
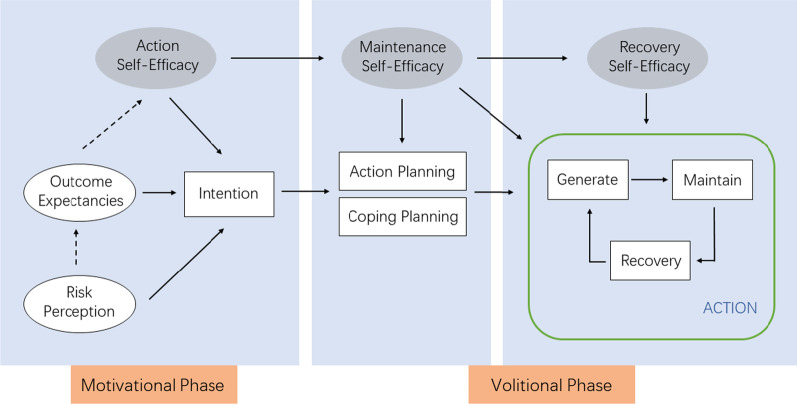
Detailed path in the HAPA model

In recent years, the health action process approach (HAPA) theory has become increasingly used in oral hygiene behavior interventions [6, 7]. This theory was first proposed by Schwarzer in 1992 [8] who stated that health behavior changes arising from a series of psychological factors and behavioral changes involve different stages. HAPA aims to promote the effective transformation from behavioral ‘intention’ in the motivational phase to specific ‘planning’ in the volitional phase. (Fig. 1). In 2008, Schwarzer proposed a detailed path design for the HAPA model based on a previous study [9]. According to the meta-analysis of Scheerman et al. [10], HAPA theory could be considered as theoretical support for existing intervention measures.

With advances on the Internet and as mobile phone functions become more comprehensive, the popularization and increased usage of mobile phones have enabled the possibility of using mobile devices to help patients develop good oral hygiene habits [11]. Smartphones can be used as a medium to effectively provide oral care knowledge, improve oral-health behavior, and oral hygiene [12, 13]. Therefore, we aimed to develop a special WeChat mini-program called ‘Clean Teeth’ in this study, which utilized HAPA theory intervention to systematically intervene in the oral hygiene behavior of patients, to enable them to develop better oral hygiene habits.

## Methods

### Study participants

This was a randomized clinical trial; 66 new patients who sought treatment from September 2020 to April 2021 at the Orthodontics Department of Anhui Stomatological Hospital met the inclusion criteria. 2 of them declined to participate in, and 20 of them recently used drugs that affect plaque attachment. 44 patients participated in. There was no loss of participants.

*Inclusion criteria* (1) Adults aged 17–29 years; (2) patients with fixed orthodontic appliances (teeth with braces at least from second left premolar to second right premolar in one arch); (3) complete dentition without edentulism, enamel and dentine dysplasia, and craniomaxillofacial defects; (4) patients without systemic and psychiatric diseases; (5) not participating in other stomatological studies; and (6) provided their informed consent.

*Exclusion criteria* (1) Recent use of drugs that affect plaque attachment, such as mouthwash use within the past month; and (2) long-term use of antibiotics.

### Experimental procedures

The participants were randomized for allocation into an experimental group and a control group, with 22 patients per group. An Internet tool (www.random.org/) was used for randomization and patients were numbered and randomized into the two groups (See Supplementary figure 1 in Additional file 1 for details). A co-author who was not involved in data collection or analysis did the randomization, and an independent researcher informed individual participants in a separate room to which group they had been allocated. If they were allocated to the intervention group, the researcher would help them unlock the mini-program on their smartphones and provide information on how to use it.

**Fig. 2:**
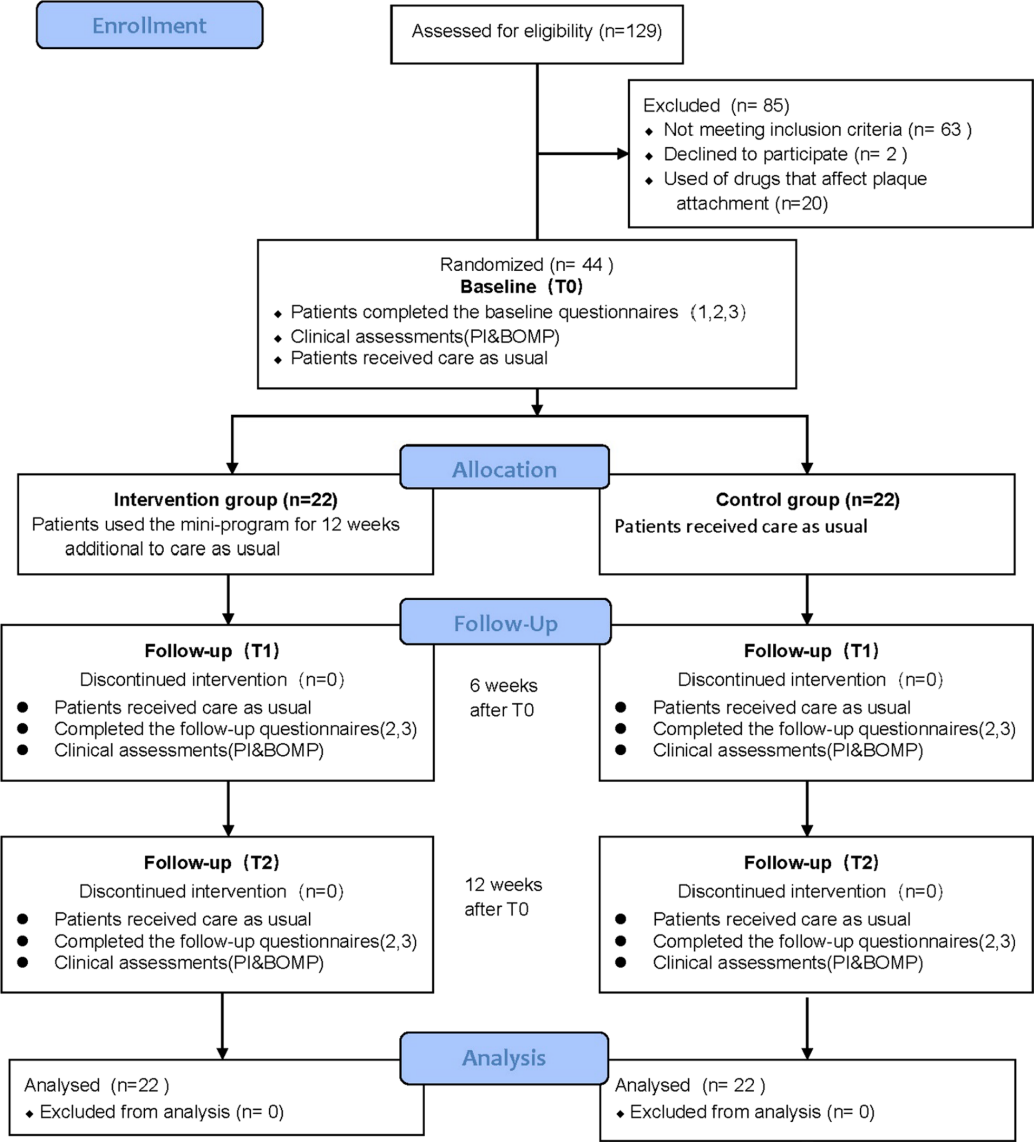
Study procedures

In the experimental group, the HAPA theory was used to provide instructions in stages combined with the mini-program. In the control group, only care as usual was provided. Markers in T0 (before treatment), T1 (after 6 weeks), and T2 (after 12 weeks) were compared to validate whether HAPA theoretical guidance had a positive clinical significance in developing good oral hygiene habits. The detailed experimental procedure was as follows (Fig. 2).

T0 Before treatment, questionnaires 1, 2, and 3 were distributed to the two groups for completion and oral hygiene education was performed. A periodontal probe (tapered tine, tip diameter 0.5 mm; DE-450, Majestic, U.K.) was used and gingival bleeding was recorded. Following that, plaque disclosing agents (brand: Y-kelin. Anhui Food and Drug Administration Medical Device Production License No. 20180015) were applied and intraoral photographs were taken. Patients in the experimental group were given disclosing agents for weekly use and required to download the mini-program. Photographs were taken and uploaded on the mini-program after using the disclosing agents.

T1: Follow-up consultation was carried out after 6 weeks, and questionnaires 2 and 3 were completed. Routine oral hygiene education was carried out and gingival bleeding and plaque disclosure status were recorded. Patients in the experimental group were asked about mini-program usage and the next cycle of the disclosing agent was distributed.
T2: Follow-up consultation was carried out after 12 weeks and the content was the same as T1. The disclosing agent would not be distributed to the patients after this phase.

### Questionnaires

The study of Scheerman [7] was used as a reference for questionnaire design. Questionnaire 1 was mainly used to collect the basic information of patients and factors that may affect oral hygiene. Questionnaire 2 was mainly used to survey oral hygiene behavior in patients. Questionnaire 3 was designed based on the detailed HAPA path model, and it included various psycho-social influencing factors in the HAPA model, such as questions designed for self-efficacy and an action plan (see attachments).

### Oral hygiene education

The oral hygiene education included routine hygiene education and plaque control guidance, such as brushing and using mouthwash and dental floss. In addition, secondary measures such as interdental brush, oral irrigator, or dental floss could be used in combination, and it was recommended that mouthwash should be used regularly.

### Clinical measurements

#### Clinical measurements were classified into two types

*Plaque index (PI)* Each tooth was divided into four regions by the bracket, namely, the gingival, mesial, distal, and occlusal regions according to the modified Silness and Loë plaque index by Williams [14]. After the disclosing agent was used, the number of regions with plaque was recorded. If one area had plaque, a score of 1 was recorded; if two areas had plaque, a score of 2 was recorded, and so on.

*Gingival bleeding* According to the method described by Van der Weijden et al. [16], A periodontal probe (tapered tine, tip diameter 0.5 mm) was inserted into the gingival pocket and run along the soft tissue wall at the orifice of the pocket at a 60 angle approximately to the longitudinal axis of the tooth, and bleeding was recorded after 30 s. If bleeding was present, a score of 1 was recorded; otherwise, if bleeding was absent, a score of 0 was recorded (four sites were examined, namely, disto-vestibular, mesio-vestibular, disto-lingual, and the mesio-lingual surfaces).

Clinical recordings were performed by two clinical examiners who underwent uniform training beforehand to increase consistency. The plaque index was scored by these two clinical examiners separately and then the average of all the scores was taken as the final score. Gingival bleeding was scored only by one examiner because of its non-repeatability.

### Blinding

The participants in this study cannot be blinded for the intervention allocation after randomization. To ensure the blindness of clinical markers, the principal researcher asked the participants not to communicate with clinical markers on whether group they are in.

### Sample size

The sample size calculation was based on the primary outcome indicator, the plaque index. It was found that the mean difference between the two groups at week 12 was 0.55 [15]. According to the following formula, the required sample size was 17 patients in each group (setting α = 0.05 and Z value was bilateral, Zα = 1.96; β was unilateral, Zβ = 1.28 at test efficacy of 0.9) [16]:

$$n = \frac{{\left( {{{Z\alpha }} + {{Z\beta }}} \right)^{2} {*}2^{2} }}{{{\updelta }^{2} }}$$

The expected loss (patient loss) was about 10%, so samples from 19 patients were required in each group. The larger the sample size, the better. Considering the experiment time and other related factors, this experiment was set as 22 participants in each group.

### Development and introduction of mini-program ‘Clean Teeth’

The ‘Clean Teeth’ mini-program was used as an additional intervention measure. This mini-program integrated the various behavioral measures based on the HAPA that were theoretically divided into the motivational phase of intention formation and the volitional phase. The measures involving periodic use of a disclosing agent, photography, toothbrushing, clocking-in through the mini-program, and video guidance for correct oral hygiene behavior were applied to the motivational phase. Additionally, measures including self-monitoring, goal setting, implementation of intention, coping planning (through volition form), and behavioral target reminders were applied to the volitional phase.

The main menu of ‘Clean Teeth’ contained a clock-in timer interface for toothbrushing and other oral-health behaviors. Related videos guiding various oral hygiene behaviors were also presented. When the patient clocked in, a relevant record would be generated to intuitively present the daily number of oral-health behaviors and the related achievements would be unlocked to encourage clocking-in.

Notifications for toothbrushing were periodically sent to patients during the 12-week intervention period. Before brushing, patients could choose whether to start the timer, and the mini-program would give a positive feedback (encouragement or achievement unlocking) when finished. In addition to the timer function, the timer interface could also provide scientific guidelines on brushing teeth to patients. If the patient did not use the mini-program, a reminder was sent every 3 days. This reminder was personalized and was based on the expected outcomes completed by the patient when they first used the mini-program. For example, “Teeth brushing can maintain oral hygiene and make your smile look better.”

Meanwhile, patients were required to use the disclosing agent every week and take three photographs of their teeth to image plaques (one photograph of the frontal occlusal surface and two photographs of the lateral occlusal surfaces). After the patient had uploaded the photographs, the orthodontist reviewed them and assessed the photographs. If the patient’s oral hygiene was poor and there was a substantial amount of disclosed plaque, the orthodontist would remind the patient to pay more attention to oral hygiene maintenance and focus on regions with most plaque in the photographs.

### Statistical analysis

SAS statistical software was used for statistical processing of the measurement results. The result of Interclass Correlation Coefficient (ICC) showed good consistency between the two researchers involved in clinical recording.

Independent sample t-tests were used to compare differences between the two groups of patients in clinical recorders and questionnaire scores at T0, T1, and T2, and differences between different phases in the same group of patients. The significance level was α = 0.05.

## Results

### Descriptive statistics

See Table 1 (See Supplementary table 1-3 in Additional file 1 for details).

**Table 1:** Statistical results of questionnaire one

	Intervention group		Control group	
Gender	Male	10	Male	11
	Female	12	Female	11
Age	18–29		18–28	
Smoking	Yes	1	Yes	0
	No	21	No	22
Toothbrush usage	Electric	4	Electric	4
	Manual	12	Manual	14
	Both of all	6	Both of all	4
Having desserts (times)	0	5	0	8
	1	10	1	10
	2	6	2	4
	> 3	1	> 3	0

### Questionnaire scores

The questionnaire was divided into 9 scales of oral health behavior, intention, self-efficacy, action plan, response plan, expected outcomes, risk perception, social effects, and behavioral control. Comparative analysis between different phases and groups was carried out for the different scales (Table 2) (See Supplementary table 4-5 in Additional file 1 for details).

**Table 2:** Statistical results of scales

Scales	Time	Intervention group	Control group	t	*P*
(a) Comparison between two groups in different periods					
Oral health behavior	T0	76.79 ± 44.02	64.20 ± 33.48	− 1.07	0.2918
	T1	126.60 ± 41.45	122.40 ± 38.53	− 0.35	0.7306
	T2	125.40 ± 50.69	121.30 ± 39.69	− 0.3	0.7671
Intention	T0	17.23 ± 2.31	16.36 ± 3.55	− 0.96	0.3454
	T1	17.23 ± 2.84	17.36 ± 3.08	0.15	0.8795
	T2	18.09 ± 1.69	17.91 ± 2.29	− 0.3	0.7657
Self-efficacy	T0	82.32 ± 10.13	79.19 ± 13.44	− 0.87	0.3875
	T1	84.23 ± 11.11	83.45 ± 11.66	− 0.23	0.823
	T2	86.36 ± 9.41	85.68 ± 9.17	− 0.24	0.8089
Action planning	T0	17.05 ± 4.71	18.00 ± 4.57	0.68	0.4985
	T1	19.27 ± 4.18	19.91 ± 3.83	0.53	0.6011
	T2	19.19 ± 4.41	20.86 ± 3.48	1.4	0.1684
Coping planning	T0	16.36 ± 5.13	16.77 ± 5.15	0.26	0.7932
	T1	18.32 ± 4.66	18.05 ± 4.72	− 0.19	0.848
	T2	18.00 ± 4.46	18.36 ± 4.49	0.27	0.7889
Outcome expectancies	T0	26.68 ± 3.18	26.95 ± 3.11	0.29	0.7751
	T1	28.45 ± 2.61	28.23 ± 2.67	− 0.29	0.7769
	T2	28.59 ± 2.68	29.09 ± 2.02	0.7	0.4894
Risk perception	T0	38.68 ± 5.39	37.27 ± 6.72	− 0.77	0.4478
	T1	37.77 ± 4.74	37.64 ± 6.42	− 0.08	0.9365
	T2	37.00 ± 5.47	39.55 ± 7.55	1.28	0.2078
Social influences	T0	30.00 ± 2.91	30.13 ± 4.69	0.12	0.9084
	T1	31.18 ± 3.17	31.05 ± 4.28	− 0.12	0.9051
	T2	31.95 ± 2.97	31.45 ± 3.83	− 0.48	0.6308
Action control	T0	24.36 ± 4.54	24.82 ± 4.20	0.34	0.7322
	T1	27.36 ± 2.95	26.95 ± 3.06	− 0.45	0.6543
	T2	27.41 ± 2.99	27.50 ± 3.16	0.1	0.9223

Questionnaires	Periods	Intervention group		Control group	
		t	*P*	t	*P*
(b) Comparison with different periods in each group					
Oral health behavior	T0–T1	− 3.86	0.0004**	− 5.35	< .0001**
	T0–T2	− 3.39	0.0015**	− 5.16	< .0001**
	T1–T2	0.08	0.9328	0.09	0.9266
Intention	T0–T1	0	1	− 1	0.3243
	T0–T2	− 1.42	0.1646	− 1.72	0.0936
	T1–T2	− 1.22	0.2275	− 0.67	0.5087
Self-efficacy	T0–T1	− 0.6	0.5548	− 1.13	0.2665
	T0–T2	− 1.37	0.1774	− 1.87	0.0688
	T1–T2	− 0.69	0.4954	− 0.7	0.4852
Action planning	T0–T1	− 1.66	0.1044	− 1.5	0.1407
	T0–T2	− 1.55	0.1279	− 2.34	0.0245*
	T1–T2	0.07	0.9444	− 0.87	0.3919
Coping planning	T0–T1	− 1.32	0.1934	− 0.85	0.3977
	T0–T2	− 1.13	0.2656	− 1.09	0.2813
	T1–T2	0.23	0.8183	− 0.23	0.8198
Outcome expectancies	T0–T1	− 2.02	0.4999	− 1.46	0.1529
	T0–T2	− 2.15	0.0375*	− 2.7	0.0104*
	T1–T2	− 0.17	0.8653	− 1.21	0.2339
Risk perception	T0–T1	0.59	0.5558	− 0.18	0.8553
	T0–T2	1.03	0.3103	− 1.05	0.2977
	T1–T2	0.5	0.6192	− 0.9	0.3713
Social influences	T0–T1	− 1.29	0.205	− 0.67	0.5058
	T0–T2	− 2.21	0.033*	− 1.02	0.3133
	T1–T2	− 0.83	0.4088	− 0.33	0.7399
Action control	T0–T1	− 0.26	0.0129*	− 1.93	0.0615
	T0–T2	− 2.63	0.0125*	− 2.39	0.0217*
	T1–T2	− 0.05	0.9598	− 0.58	0.564

*P < 0.05, **P < 0.01

### Clinical test scores

Scores were obtained for plaque and gingival bleeding (Table 3) (See Supplementary table 6-7 in Additional file 1 for details).

**Table 3:** Statistical results of clinical assessments

Time	Intervention group	Control group	t	*P*	Period	Intervention group		Control group	
						t	*P*	t	*P*
(a) Dental plaque									
T0	0.39 ± 0.28	0.55 ± 0.49	1.34	0.1872	T0–T1	− 0.37	0.0007**	− 6.71	< 0.0001**
T1	0.81 ± 0.46	1.61 ± 0.56	5.15	< 0.0001**	T0–T2	− 3.93	0.0003**	− 5.24	< 0.0001**
T2	0.88 ± 0.51	1.34 ± 0.52	2.96	0.005**	T1–T2	− 0.47	0.6441	1.63	0.11

Time	Intervention group	Control group	t	*P*	Period	Intervention group		Control group	
						t	P	t	*P*
(b) Gingival bleeding									
T0	0.49 ± 0.42	0.55 ± 0.67	0.97	0.3399	T0–T1	1.12	0.0373*	− 0.58	0.5627
T1	0.37 ± 0.57	0.65 ± 0.42	1.85	0.0425*	T0–T2	1.13	0.0305*	− 0.45	0.6585
T2	0.37 ± 0.53	0.63 ± 0.55	1.62	0.0831	T1–T2	0	0.9979	0.11	0.9138

*P < 0.05, **P < 0.01

### Number of people with additional oral hygiene measures

**Fig. 3:**
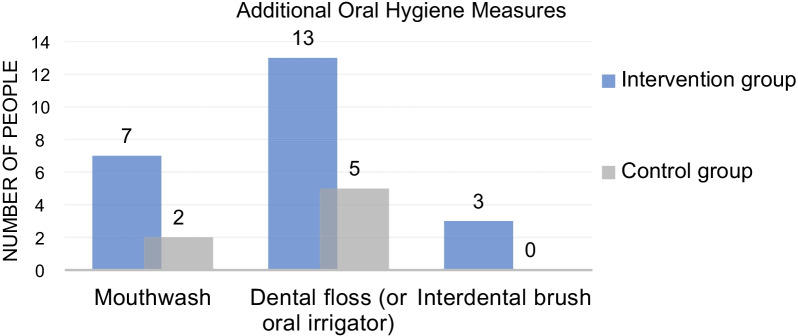
Additional oral hygiene measures

Pre-treatment routine oral-health behavior in most patients only involved tooth brushing. However, it was found that some patients added flossing, interdental brushing, or gargling in their routine during treatment. Among the patients, gargling, flossing or oral irrigator, and interdental brushing were added for 7, 13, and 3 subjects, respectively, in the experimental group; and 2, 5, and 0 subjects, respectively, in the control group. (Fig. 3).

## Discussion

The HAPA model was first proposed by Schwarzer [8] and has been currently widely used in many fields, such as measuring social distancing behavior [17], physical activity [18, 19], dieting [20], depression prevention [21], and oral hygiene [7, 18, 22]. This is due to the theoretical strengths and widespread use of the HAPA model. However, there are limited studies on oral hygiene behavior in patients with fixed orthodontic appliances. In this study, HAPA theory was used as a basis to develop a WeChat mini-program for menu-based interventions [6] (i.e., a set of predesigned measures or methods was immediately implemented for different intervention subjects) and relevant questionnaires were designed to achieve effective intervention in oral-health behavior in patients. In particular, the usage rate of mobile phones is relatively high in young adults and WeChat is a basic essential app that is commonly installed, with young adults being therefore selected as the research object in this study. A mini-program developed based on HAPA theory was used to monitor daily oral-health habits and to intervene in oral health behaviors, and its application in the field of orthodontics was evaluated.

The mini-program ‘Clean teeth’, as an intervention measure, acts on two major phases of the motivational phase and the volitional phase. Behaviors in the motivational phase included watching videos about oral hygiene and taking photos of teeth and uploading on the mini-program weekly, which strengthened the patient's oral health awareness, namely the "intention" factor. The volitional phase involved clocking in a mini-program after teeth brushing or other oral cleaning measure. The more the patients clocked in, the more achievements were input, which exerted positive feedback. If the patients showed too much plaque, the orthodontists were able to remind them based on their uploaded pictures. Furthermore, the mini-program had a function of sending personalized reminder messages if patients had not logged in for a long time.

Social psychology studies have shown that the mean duration required for a behavior to become a habit is 66 days, but there are differences in the time needed for different behaviors owing to the different complexities [23]. This study was focused on toothbrushing and other oral hygiene behavior in patients. This behavioral habit was basically formed before orthodontic treatment, and the aim was to strengthen this habit so that patients with insufficient toothbrushing duration or frequency could meet the requirements. Therefore, the study duration was designed to span 12 weeks. The results of this study showed that oral hygiene behavior and periodontal clinical examinations were relatively satisfactory. However, further follow-up observations are still required for assessment after week 12.

In this study, the basic information of patients was collected in questionnaire 1. Both groups were adult patients under 30 years old, with a balanced gender ratio. Questionnaire 2 was an oral-health behavior survey conducted in patients. The larger the number, the more frequent or more diverse the oral-health behavior was. According to the statistical results, there were obvious increases in T1 and T2 scores as compared with the T0 score in the experimental and control groups, and these differences were statistically significant. This shows that the frequency or duration of oral-health behaviors, such as toothbrushing, gargling, and flossing was effectively increased after orthodontic devices were fitted on patients. The mean values of the experimental group were greater than that of the control group, but the differences were not statistically significant. There were no statistically significant changes in scores from T1 to T2 in both groups, showing that as wear duration increased, there were few changes in oral-health behavior. In addition, as can be seen from the questionnaire results, other than toothbrushing, the proportion of people with additional oral-health measures was double in the experimental group compared with the control group. This showed that the oral-health behavioral changes were more significant in the experimental group compared to the control group.

Questionnaire 3 of this research included various factors in the HAPA model to understand and analyze psychosocial factors of patients during treatment. According to the statistical results, changes in most psychosocial scale scores were not significant during the 3-month trial, and only expected outcomes, social effects, and action control showed statistically significant differences before and after treatment.

In “expected outcomes,” the scores in the experimental group and control group increased from T0 to T2, and these differences were statistically significant. This showed that both groups had an optimistic attitude towards their own oral hygiene status during orthodontic treatment. On one hand, this may be due to encouragement provided by medical staff during oral health education. On the other hand, the confidence of patients increased due to significant increase in toothbrushing and other oral-health habits after wearing their orthodontic appliances.

As for “social effects,” only the increase in scores of the experimental group from T0 to T2 was statistically significant, and there were no significant changes in the control group. This means that mini-program usage may be beneficial towards social interaction in oral hygiene because the social interactions of young people are intimately related to mobile phones.

With regards to “action control,” there were significant differences from T0 to T1 and from T0 to T2 in the experimental group and from T0 to T2 in the control group, showing that both groups of patients implemented self-discipline in oral hygiene behavior during treatment. Among these groups, the experimental group started showing significant changes from T1 onward and the control group only started showing significant changes from T2 onward, showing that mini-program intervention had positive effects because it can induce self-discipline in patients and that was maintained until T2.

In clinical measurements, the modified Silness and Loë plaque index by Williams [15] was used to score plaque, and gingival bleeding was assessed with the Bleeding on Marginal Probing index (BOMP) [16]. A systematic review [14] concluded that the modified Silness and Loë plaque index by Williams [15] was the most valid and discriminatory index to score plaque in patients with fixed appliances. We used the same method to divide each tooth into four regions by the bracket, and points were scored according to the number of areas the plaque shown on the teeth after using plaque disclosing agents.

Gingival bleeding was assessed with the Bleeding on Marginal Probing index (BOMP). Van et al. [16] found Angulated bleeding index [26, 27] (that the probe was inserted and run along the marginal gingiva and was held at an angle of approximately 60° to the longitudinal axis of the tooth) was more accurately to evaluate teeth with a healthy gingival condition than the Parallel bleeding index [27](when the probe was run along the marginal gingiva it was held parallel to the longitudinal axis of the tooth). Therefore, we selected Angulated bleeding index.

From the clinical examination results, it was found that there was no significant difference in the T0 plaque index scores between the experimental group versus control group, validating that the patients were randomly assigned and pre-treatment status was consistent. After treatment began, there were significant differences between the T1 and T2 phases, and scores of the experimental group were lower than those of the control group. This shows that the amount of plaque in the experimental group decreased significantly compared with that of the control group. The two groups were compared based on different periods. The results showed that there were significant differences from T0 to T1 and from T0 to T2, with T1 and T2 values being both higher than T0, showing that the plaque index increased after orthodontic devices were fitted to patients in both groups. This revealed that wearing a fixed orthodontic appliance would still significantly increase the amount of dental plaque even though oral hygiene interventions were carried out. However, increased plaque in the experimental group was significantly reduced compared with the control group after the mini-program intervention was carried out. Alkadhi, O. H [24]. found that the app had reduced the dental plaque more effectively after the intervention than verbal oral-hygiene instructions, while some studies [13, 25] found no significant difference between the groups.

The gingival bleeding index results indicated that differences between the two groups were not large before treatment but differences were still statistically significant in T1, showing that the bleeding index was reduced in the experimental versus control group. According to the mean values at different time periods, the bleeding index decreased after T0 in the experimental group, and this difference was statistically significant. There was a slight increase in the control group, but this difference was not statistically significant at the threshold of P < 0.05 level. Overall, the experimental group showed better performance in gingival bleeding than the control group, with a gradual declining trend. Our results were similar to those of previous studies. Homa Farhadifard et al. [13] found that the gingival bleeding index changes between the two groups (*P* < 0.001). Meanwhile, a reduction both in gingival bleeding index and plaque index in the intervention group was noted in comparison with the control group. Deleuse, M. et al. [25] also found that gingival bleeding index decreased significantly in the intervention group over the 4 weeks of the study.

The limitations of this study were that there was only a relatively small sample size. The study duration could be further extended to assess whether patients continued to maintain habits that were previously developed. Furthermore, mini-program functions can be further improved in the future, such as superimposing gridlines over the photos that patients uploaded, these can compute the plaque score for every tooth by themselves, based on the colorimetric results. Giving deeper feedback based on the score also needs to be optimized. It is hoped that the study duration and scope can be further expanded, and further updates and improvements can be made based on the user feedback regarding the mini-program.

## Conclusions

Overall, the amount of plaque in the two groups increased significantly during treatment with fixed orthodontic appliances. However, the plaque index in the experimental group was significantly lower than that in the control group, showing that mini-program intervention had significant effects on plaque control. In addition, the gingival bleeding index was not increased in the two groups while being significantly decreased in the experimental group.

The psycho-social parameters of social effects, expected outcomes, and action control were significantly increased after treatment, among which social effects increased significantly only in the experimental group. This thus shows that mini-program usage may be beneficial for social interaction.

Hence, it can be concluded that the HAPA theory-based mini-program had significant effects in improving oral-health behavior and oral-hygiene outcomes among patients. A larger sample size is required for further study to promote this mini-program in clinical practice.

## Supplementary Information

**Additional file 1.** Raw data.

## Data Availability

All data generated or analysed during this study are included in this published article and its Additional files.

## Abbreviations

HAPA: Health action process approach
PI: Plaque index
