# Supplementary material for: Effectiveness of an online application of the health action process approach (HAPA) theory on oral hygiene intervention in young adults with fixed orthodontic appliances: a randomized controlled trial
Source: BMC Oral Health. 2022 May 19;22:192. doi: 10.1186/s12903-022-02219-w (PMC9118762; doi:10.1186/s12903-022-02219-w)
Supplement: Supplementary file 1 — Additional file 1. Raw data. [file 12903_2022_2219_MOESM1_ESM.docx]

**Supplementary table 1.** Patient numbering and grouping

| Intervention group | | Control group | |
| --- | --- | --- | --- |
| Serial number | Patient's name | Serial number | Patient's name |
| 2 | Yalan Wang | 1 | Yating Liang |
| 3 | Hanling Xu | 5 | Wenjuan Zhao |
| 4 | Niannian Yu | 6 | Changcai Ge |
| 7 | Julong Song | 8 | Xiaoli Yang |
| 10 | Yanping Fang | 9 | Mingzhu Yao |
| 13 | Liu Chen | 11 | Rong Fu |
| 14 | Jun Hu | 12 | Xiaoyun Lu |
| 15 | Jie Wang | 16 | Wenjing Cui |
| 17 | Xiaoli Wang | 21 | Weina Hu |
| 18 | Ziyi Fan | 22 | Lei Du |
| 19 | Chengbo Hu | 23 | Chao Zhang |
| 20 | Yangyang Lu | 24 | Qingyun Liu |
| 25 | Gui Tan | 26 | Mengying Xu |
| 27 | Guoqing Xi | 30 | Kunpeng Ning |
| 28 | Fanghui Lin | 31 | Shuai Jiang |
| 29 | Yanbo Yang | 32 | Haiwang Zhang |
| 33 | Xingyu Zhang | 37 | Gang Liu |
| 34 | Qian You | 40 | Pengfei Jiang |
| 35 | Yang Lv | 41 | Ziyang Xia |
| 36 | Xuhui Zhao | 42 | Minglun Ni |
| 38 | Jianing Zhao | 43 | Taoyun Ye |
| 39 | Min Wang | 44 | Jie Wang |


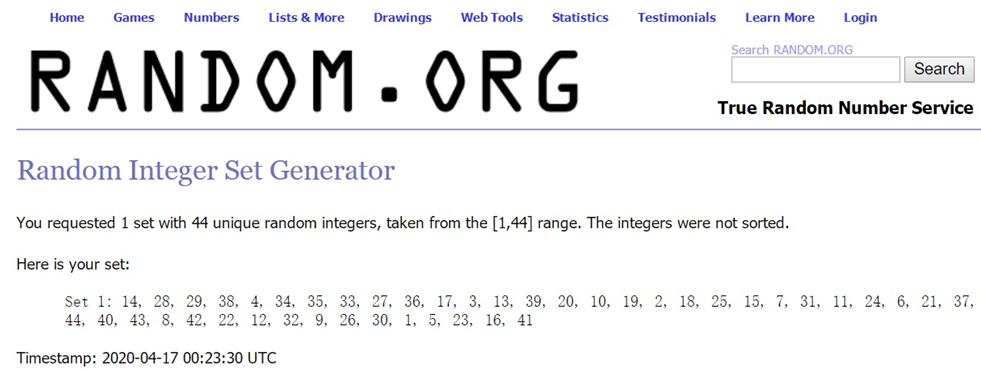


**Supplementary figure 1.** Randomization

**Supplementary table 2.** Questionnaire one

| 1 | Gender | | | | | |
| --- | --- | --- | --- | --- | --- | --- |
|  | 0 1 | Male  Female | | | | |
|  | | | | | | |
| 2 | Age |  | Birthdate |  | | |
|  | | | | | | |
| 3 | Educational background | | | | | |
|  | 1 2 3 4 5 | High school degree  Junior college degree  Bachelor  Master  Doctoral degree | | | | |
|  | | | | | | |
| 4 | Smoking | | | | | |
|  | 1 0 | Yes  No | | | | |
|  | | | | | | |
| 5 | Toothbrush usage | | | | | |
|  | 1  2  3 | electric  manual  both of all | | | | |
|  | | | | | | |
| 6 | Do you eat sugary foods or drinks after meals? What is the number of times? | | | | |  |
|  | Instructions: 1) If you eat sugary food and drink sugary drinks at the same time, the count is 1; If there is more than an hour between eating sugary food and drinking the sugary, the count is 2; 2) It must be sugary food or drink, for example, unsweetened coffee is not counted, but sugary coffee is. | | | |  |  |

**Supplementary table 3.** Raw data of questionnaire one

| Serial number of patients | Gender | Age | Educational background | Smoking | Toothbrush usage | Having desserts (times) |
| --- | --- | --- | --- | --- | --- | --- |
| 1 | 1 | 21 | 3 | 0 | 2 | 0 |
| 2 | 1 | 29 | 3 | 0 | 3 | 1 |
| 3 | 1 | 19 | 2 | 0 | 1 | 2 |
| 4 | 1 | 22 | 3 | 0 | 2 | 1 |
| 5 | 1 | 27 | 4 | 0 | 2 | 0 |
| 6 | 0 | 25 | 3 | 0 | 1 | 0 |
| 7 | 0 | 18 | 2 | 0 | 2 | 1 |
| 8 | 1 | 24 | 3 | 0 | 2 | 0 |
| 9 | 1 | 26 | 3 | 0 | 3 | 1 |
| 10 | 1 | 24 | 2 | 0 | 1 | 1 |
| 11 | 1 | 27 | 3 | 0 | 1 | 1 |
| 12 | 1 | 27 | 3 | 0 | 3 | 1 |
| 13 | 1 | 20 | 3 | 0 | 3 | 0 |
| 14 | 0 | 25 | 5 | 0 | 1 | 0 |
| 15 | 0 | 24 | 3 | 0 | 2 | 1 |
| 16 | 1 | 21 | 2 | 0 | 2 | 2 |
| 17 | 1 | 36 | 3 | 0 | 2 | 0 |
| 18 | 1 | 19 | 3 | 0 | 2 | 1 |
| 19 | 0 | 27 | 2 | 0 | 3 | 0 |
| 20 | 0 | 19 | 3 | 0 | 2 | 1 |
| 21 | 1 | 26 | 3 | 0 | 3 | 2 |
| 22 | 0 | 28 | 2 | 0 | 2 | 1 |
| 23 | 1 | 27 | 2 | 0 | 2 | 1 |
| 24 | 1 | 24 | 2 | 0 | 2 | 1 |
| 25 | 1 | 22 | 2 | 0 | 3 | 2 |
| 26 | 1 | 17 | 2 | 0 | 2 | 1 |
| 27 | 0 | 25 | 4 | 0 | 1 | 2 |
| 28 | 1 | 24 | 4 | 0 | 2 | 2 |
| 29 | 1 | 32 | 1 | 0 | 2 | 1 |
| 30 | 0 | 18 | 2 | 0 | 2 | 0 |
| 31 | 0 | 20 | 2 | 0 | 2 | 2 |
| 32 | 0 | 18 | 3 | 0 | 2 | 1 |
| 33 | 0 | 19 | 2 | 0 | 2 | 1 |
| 34 | 1 | 28 | 2 | 0 | 3 | 0 |
| 35 | 0 | 29 | 4 | 0 | 2 | 1 |
| 36 | 0 | 23 | 3 | 1 | 3 | 2 |
| 37 | 0 | 31 | 3 | 0 | 2 | 0 |
| 38 | 0 | 18 | 3 | 0 | 2 | 3 |
| 39 | 1 | 28 | 2 | 0 | 2 | 2 |
| 40 | 0 | 16 | 1 | 0 | 1 | 2 |
| 41 | 0 | 19 | 3 | 0 | 2 | 1 |
| 42 | 0 | 20 | 3 | 0 | 3 | 0 |
| 43 | 0 | 18 | 3 | 0 | 2 | 1 |
| 44 | 0 | 21 | 3 | 0 | 1 | 0 |

**Supplementary table 4**. Questionnaire two and three

| The following questions are related to your oral health, please choose according to your personal situation. Thank you very much for your participation in filling out these questionnaires! | | | | | | | | | | | | | | | | | | |
| --- | --- | --- | --- | --- | --- | --- | --- | --- | --- | --- | --- | --- | --- | --- | --- | --- | --- | --- |
| How often did the following oral health care measures occur in the past 4 weeks? | | | | | | | | | | | | | | | | | | |
|  | | ≥3 times/day | | 2 times/day | | 1 time/day | 2~3 times/week | | | | 1 time/week | | | | | 2 times/month | Very little/Never | |
| 1 | Toothbrushing |  | |  | |  |  | | | |  | | | | |  |  | |
| 2 | Interdental brushes |  | |  | |  |  | | | |  | | | | |  |  | |
| 3 | Toothpick |  | |  | |  |  | | | |  | | | | |  |  | |
| 4 | Mouth rinse |  | |  | |  |  | | | |  | | | | |  |  | |
| 5 | Other measures (Please give examples) |  | |  | |  |  | | | |  | | | | |  |  | |
| 6 | How long did you brush your teeth each time? | | | | | | | | | | | min | | | | | | |
|  | | | | | | | | | | | | | | | | | | |
| How many times did you do the following oral health care measures yesterday? | | | | | | | | | | | | | | | | | | |
|  | | ≥3 times | | | | 2 times | | | | | 1 time | | | | | | None | |
| 7 | Toothbrushing |  | | | |  | | | | |  | | | | | |  | |
| 8 | Interdental brushes |  | | | |  | | | | |  | | | | | |  | |
| 9 | Mouth rinse |  | | | |  | | | | |  | | | | | |  | |
|  | | | | | | | | | | | | | | | | | | |
| How often did the following oral health care measures occur in the past 4 weeks? | | | | | | | | | | | | | | | | | | |
| 10 | Toothbrushing | | | | | | | | | | times/day | | | | | | | |
| 11 | Interdental brushes | | | | | | | | | | times/day | | | | | | | |
| 12 | Mouth rinse | | | | | | | | | | times/day | | | | | | | |
|  | | | | | | | | | | | | | | | | | | |
| If you use a interdental brush, please answer the following questions. If not, please skip it. | | | | | | | | | | | | | | | | | | |
| How do you use a interdental brush? | | | | | | | | | | | | | | | | | | |
|  | | | | | | | Yes | | | | | | | | | No | | |
| 11b | Clean between teeth | | | | | |  | | | | | | | | |  | | |
| 11c | Clean between brackets | | | | | |  | | | | | | | | |  | | |
|  | | | | | | | | | | | | | | | | | | |
| In the next four weeks, I plan to... | | | | | | | | | | | | | | | | | | |
|  | | | | Totally disagree | | | | Disagree | | | Not sure | | | | Agree | | Totally agree | |
| 13a | Brush my teeth at least twice a day. | | |  | | | |  | | |  | | | |  | |  | |
| 13b | Brush for at least 3 minutes each time. | | |  | | | |  | | |  | | | |  | |  | |
| 14a | Clean my teeth daily with a interdental brush. | | |  | | | |  | | |  | | | |  | |  | |
| 14b | Use mouth rinse every day. | | |  | | | |  | | |  | | | |  | |  | |
|  | | | | | | | | | | | | | | | | | | |
| I am sure that I can... | | | | | | | | | | | | | | | | | | |
|  | | | | Totally disagree | | | | Disagree | | | Not sure | | | | Agree | | Totally agree | |
| 15 | Brush for no less than 3 minutes each time. | | |  | | | |  | | |  | | | |  | |  | |
| 16 | Brush my teeth at least twice a day. | | |  | | | |  | | |  | | | |  | |  | |
| 17 | Brush my teeth carefully everyday. | | |  | | | |  | | |  | | | |  | |  | |
| 18 | Brush my teeth very carefully everyday, even the dental surfaces that are difficult to brush. | | |  | | | |  | | |  | | | |  | |  | |
| 19 | Use a interdental brush daily. | | |  | | | |  | | |  | | | |  | |  | |
| 20 | Use a interdental brush carefully everyday. | | |  | | | |  | | |  | | | |  | |  | |
| 21 | Use a interdental brush very carefully everyday, even the dental surfaces that are difficult to brush. | | |  | | | |  | | |  | | | |  | |  | |
| 21b | Use mouth rinse every day. | | |  | | | |  | | |  | | | |  | |  | |
|  | | | | | | | | | | | | | | | | | | |
| I'm sure that I can brush my teeth at least 2 times a day for more than 3 minutes each time, ... | | | | | | | | | | | | | | | | | | |
|  | | | | Totally disagree | | | | Disagree | | | Not sure | | | | Agree | | Totally agree | |
| 22 | Even though I can't immediately see the benefits of brushing. | | |  | | | |  | | |  | | | |  | |  | |
| 23 | Even if I don't like brushing my teeth. | | |  | | | |  | | |  | | | |  | |  | |
| 24 | Even though I am busy. | | |  | | | |  | | |  | | | |  | |  | |
| 25 | Even if brushing my teeth is time-consuming. | | |  | | | |  | | |  | | | |  | |  | |
|  | | | | | | | | | | | | | | | | | | |
| I'm sure I can clean my teeth everyday with a interdental brush, ... | | | | | | | | | | | | | | | | | | |
|  | | | | Totally disagree | | | | Disagree | | | Not sure | | | | Agree | | Totally agree | |
| 26 | Even though I can't immediately see the benefits of a interdental brush. | | |  | | | |  | | |  | | | |  | |  | |
| 27 | Even though I don't like using a interdental brush. | | |  | | | |  | | |  | | | |  | |  | |
| 28 | Even though I am busy. | | |  | | | |  | | |  | | | |  | |  | |
| 29 | Even if it's very time-consuming. | | |  | | | |  | | |  | | | |  | |  | |
|  | | | | | | | | | | | | | | | | | | |
| I'm sure I'll be able to... for a long time to come. | | | | | | | | | | | | | | | | | | |
|  | | | | Totally disagree | | | | Disagree | | | Not sure | | | | Agree | | Totally agree | |
| 30a | Brush my teeth at least twice a day. | | |  | | | |  | | |  | | | |  | |  | |
| 30b | Brush for at least 3 minutes each time. | | |  | | | |  | | |  | | | |  | |  | |
| 31 | Clean my teeth daily with a interdental brush. | | |  | | | |  | | |  | | | |  | |  | |
|  | | | | | | | | | | | | | | | | | | |
| Do you have a plan for the following aspects? | | | | | | | | | | | | | | | | | | |
|  | | | | | | No plan | | | Vague plan | | | | Clear plan | | | | Precise plan | |
| 32 | When do I brush my teeth? | | | | |  | | |  | | | |  | | | |  | |
| 33 | Where do I brush my teeth? | | | | |  | | |  | | | |  | | | |  | |
| 34 | How much time do I spend brushing my teeth? | | | | |  | | |  | | | |  | | | |  | |
| 35 | When should I use a interdental brush? | | | | |  | | |  | | | |  | | | |  | |
| 36 | Where will I use a interdental brush? | | | | |  | | |  | | | |  | | | |  | |
| 37 | How much time do I spend using a interdental brush? | | | | |  | | |  | | | |  | | | |  | |
|  | | | | | | | | | | | | | | | | | | |
| Do you have a plan for dealing with the following situations? | | | | | | | | | | | | | | | | | | |
|  | | | | | | No plan | | | Vague plan | | | | Clear plan | | | | Precise plan | |
| 38 | Something is stopping me from brushing my teeth. | | | | |  | | |  | | | |  | | | |  | |
| 39 | I forgot to brush my teeth. | | | | |  | | |  | | | |  | | | |  | |
| 40 | Something is getting in the way of the interdental brush. | | | | |  | | |  | | | |  | | | |  | |
| 41 | I forgot to use a interdental brush. | | | | |  | | |  | | | |  | | | |  | |
|  | | | | | | | | | | | | | | | | | | |
| Do you have a plan to motivate yourself when you don't want to do the following things? | | | | | | | | | | | | | | | | | | |
|  | | | | | | No plan | | | Vague plan | | | | Clear plan | | | | Precise plan | |
| 42 | Brushing teeth | | | | |  | | |  | | | |  | | | |  | |
| 43 | Using a interdental brush | | | | |  | | |  | | | |  | | | |  | |
|  | | | | | | | | | | | | | | | | | | |
| Brushing teeth regularly can ... | | | | | | | | | | | | | | | | | | |
|  | | | | Totally disagree | | | | Disagree | | | Not sure | | | | Agree | | Totally agree | |
| 44 | Prevent tooth decay | | |  | | | |  | | |  | | | |  | |  | |
| 45 | Fresh my breath | | |  | | | |  | | |  | | | |  | |  | |
| 46 | Avoid tooth staining | | |  | | | |  | | |  | | | |  | |  | |
| 47 | Make me feel good | | |  | | | |  | | |  | | | |  | |  | |
| 48 | Promote gum health | | |  | | | |  | | |  | | | |  | |  | |
| 49 | Make me better | | |  | | | |  | | |  | | | |  | |  | |
|  | | | | | | | | | | | | | | | | | | |
| How likely do you think you are to be at risk for the following diseases? | | | | | | | | | | | | | | | | | | |
|  | | | Very low | | Low | | | | | General | | | | High | | | | Very high |
| 50 | Tooth decay | |  | |  | | | | |  | | | |  | | | |  |
| 51 | Gingivitis | |  | |  | | | | |  | | | |  | | | |  |
|  | | | | | | | | | | | | | | | | | | |
| Compared to your friends, how likely do you think you are to be at risk for the following diseases? | | | | | | | | | | | | | | | | | | |
|  | | | Very low | | Low | | | | | General | | | | High | | | | Very high |
| 52 | Tooth decay | |  | |  | | | | |  | | | |  | | | |  |
| 53 | Gingivitis | |  | |  | | | | |  | | | |  | | | |  |
|  | | | | | | | | | | | | | | | | | | |
| If I don’t brush my teeth regularly, the risk of acquiring the following diseases will be... | | | | | | | | | | | | | | | | | | |
|  | | | Very low | | Low | | | | | General | | | | High | | | | Very high |
| 54 | Tooth decay | |  | |  | | | | |  | | | |  | | | |  |
| 55 | Gingivitis | |  | |  | | | | |  | | | |  | | | |  |
| 56 | Periodontitis | |  | |  | | | | |  | | | |  | | | |  |
|  | | | | | | | | | | | | | | | | | | |
| If I don't use interdental brushes regularly, the risk of acquiring the following diseases will be... | | | | | | | | | | | | | | | | | | |
|  | | | Very low | | Low | | | | | General | | | | High | | | | Very high |
| 57 | Tooth decay | |  | |  | | | | |  | | | |  | | | |  |
| 58 | Gingivitis | |  | |  | | | | |  | | | |  | | | |  |
| 59 | Periodontitis | |  | |  | | | | |  | | | |  | | | |  |
|  | | | | | | | | | | | | | | | | | | |
| About parents | | | | | | | | | | | | | | | | | | |
|  | | | | Totally disagree | | | | Disagree | | | Not sure | | | | Agree | | Totally agree | |
| 60 | My parents often remind me to brush my teeth properly. | | |  | | | |  | | |  | | | |  | |  | |
| 61 | My parents brush their teeth at least twice a day. | | |  | | | |  | | |  | | | |  | |  | |
|  | | | | | | | | | | | | | | | | | | |
| About other peers who also accept orthodontic treatments | | | | | | | | | | | | | | | | | | |
|  | | | | Totally disagree | | | | Disagree | | | Not sure | | | | Agree | | Totally agree | |
| 62 | I believe they brush their teeth at least twice a day for at least three minutes each time. | | |  | | | |  | | |  | | | |  | |  | |
| 63 | I believe they use a interdental brush everyday to clean their teeth. | | |  | | | |  | | |  | | | |  | |  | |
|  | | | | | | | | | | | | | | | | | | |
| Who thought I should brush my teeth properly? | | | | | | | | | | | | | | | | | | |
|  | | | | Totally disagree | | | | Disagree | | | Not sure | | | | Agree | | Totally agree | |
| 64 | My friends | | |  | | | |  | | |  | | | |  | |  | |
| 65 | My doctors | | |  | | | |  | | |  | | | |  | |  | |
| 66 | My parents or guardians | | |  | | | |  | | |  | | | |  | |  | |
|  | | | | | | | | | | | | | | | | | | |
| In the last week, ... | | | | | | | | | | | | | | | | | | |
|  | | | | Totally disagree | | | | Disagree | | | Not sure | | | | Agree | | Totally agree | |
| 67 | I have started a regular check on my brushing frequency. | | |  | | | |  | | |  | | | |  | |  | |
| 68 | I've started a routine check on my brushing time. | | |  | | | |  | | |  | | | |  | |  | |
| 69 | I've already started a routine check on how often I use a interdental brush. | | |  | | | |  | | |  | | | |  | |  | |
| 70a | I've tried to brush my teeth at least twice a day. | | |  | | | |  | | |  | | | |  | |  | |
| 70b | I've been trying to brush my teeth for at least three minutes everytime. | | |  | | | |  | | |  | | | |  | |  | |
| 71 | I've tried to use a interdental brush every day. | | |  | | | |  | | |  | | | |  | |  | |

**Supplementary table 5.** Raw data of questionnaire two and three

| Serial number of patients | Phase | Oral health behavior | Intention | Self-efficacy | Action planning | Coping planning | Outcome expectancies | Risk perception | Social influences | Action control | total points |
| --- | --- | --- | --- | --- | --- | --- | --- | --- | --- | --- | --- |
| 1 | T0 | 59 | 16 | 68 | 17 | 14 | 25 | 33 | 25 | 23 | 280 |
|  | T1 | 74.5 | 17 | 72 | 16 | 14 | 26 | 38 | 26 | 25 | 308.5 |
|  | T2 | 96.5 | 16 | 82 | 16 | 14 | 26 | 42 | 26 | 26 | 344.5 |
| 2 | T0 | 76.95 | 16 | 72 | 11 | 11 | 24 | 40 | 28 | 21 | 299.95 |
|  | T1 | 104 | 16 | 78 | 15 | 15 | 30 | 41 | 34 | 28 | 361 |
|  | T2 | 154 | 18 | 93 | 24 | 24 | 30 | 40 | 35 | 30 | 448 |
| 3 | T0 | 44 | 14 | 65 | 11 | 10 | 20 | 43 | 30 | 17 | 254 |
|  | T1 | 93.45 | 18 | 93 | 21 | 16 | 30 | 42 | 33 | 24 | 370.45 |
|  | T2 | 75.5 | 17 | 80 | 18 | 18 | 30 | 44 | 33 | 24 | 339.5 |
| 4 | T0 | 168 | 16 | 76 | 16 | 13 | 24 | 38 | 28 | 24 | 403 |
|  | T1 | 206 | 16 | 76 | 18 | 16 | 24 | 33 | 28 | 24 | 441 |
|  | T2 | 194.5 | 16 | 76 | 18 | 18 | 24 | 36 | 28 | 24 | 434.5 |
| 5 | T0 | 184 | 18 | 78 | 24 | 18 | 24 | 28 | 26 | 24 | 424 |
|  | T1 | 154 | 18 | 90 | 23 | 22 | 30 | 28 | 26 | 30 | 421 |
|  | T2 | 134.5 | 17 | 83 | 19 | 18 | 30 | 32 | 26 | 28 | 387.5 |
| 6 | T0 | 58 | 18 | 74 | 11 | 8 | 30 | 42 | 31 | 20 | 292 |
|  | T1 | 132 | 16 | 87 | 18 | 14 | 30 | 31 | 33 | 30 | 391 |
|  | T2 | 114.5 | 20 | 84 | 18 | 13 | 30 | 37 | 28 | 30 | 374.5 |
| 7 | T0 | 60.5 | 14 | 67 | 15 | 16 | 24 | 30 | 28 | 22 | 276.5 |
|  | T1 | 51 | 8 | 56 | 15 | 16 | 24 | 36 | 26 | 23 | 255 |
|  | T2 | 53 | 16 | 83 | 15 | 15 | 24 | 36 | 27 | 22 | 291 |
| 8 | T0 | 63.5 | 15 | 69 | 16 | 12 | 22 | 33 | 27 | 20 | 277.5 |
|  | T1 | 86.5 | 14 | 74 | 18 | 18 | 23 | 38 | 28 | 23 | 322.5 |
|  | T2 | 90 | 15 | 71 | 18 | 12 | 24 | 37 | 28 | 23 | 318 |
| 9 | T0 | 60 | 20 | 95 | 13 | 13 | 30 | 50 | 35 | 30 | 346 |
|  | T1 | 183 | 20 | 95 | 24 | 20 | 30 | 46 | 35 | 30 | 483 |
|  | T2 | 173.5 | 20 | 95 | 24 | 24 | 30 | 44 | 35 | 30 | 475.5 |
| 10 | T0 | 44 | 20 | 92 | 15 | 22 | 24 | 40 | 33 | 24 | 314 |
|  | T1 | 191 | 20 | 95 | 21 | 20 | 30 | 40 | 31 | 30 | 478 |
|  | T2 | 183 | 20 | 95 | 20 | 20 | 30 | 38 | 32 | 30 | 468 |
| 11 | T0 | 60.95 | 16 | 76 | 20 | 18 | 27 | 30 | 30 | 24 | 301.95 |
|  | T1 | 81.45 | 16 | 73 | 17 | 16 | 24 | 36 | 28 | 24 | 315.45 |
|  | T2 | 95.45 | 15 | 76 | 18 | 18 | 30 | 35 | 28 | 23 | 338.45 |
| 12 | T0 | 51 | 20 | 92 | 15 | 20 | 25 | 41 | 29 | 28 | 321 |
|  | T1 | 128.5 | 20 | 95 | 24 | 24 | 30 | 45 | 35 | 30 | 431.5 |
|  | T2 | 135.5 | 20 | 95 | 24 | 24 | 30 | 50 | 33 | 30 | 441.5 |
| 13 | T0 | 129 | 18 | 95 | 24 | 24 | 30 | 48 | 33 | 30 | 431 |
|  | T1 | 136.5 | 20 | 95 | 24 | 24 | 30 | 40 | 31 | 30 | 430.5 |
|  | T2 | 134 | 20 | 95 | 24 | 24 | 30 | 50 | 35 | 30 | 442 |
| 14 | T0 | 83.5 | 20 | 95 | 24 | 15 | 30 | 37 | 28 | 20 | 352.5 |
|  | T1 | 165 | 20 | 95 | 24 | 24 | 30 | 34 | 31 | 30 | 453 |
|  | T2 | 164 | 20 | 95 | 24 | 18 | 30 | 38 | 29 | 30 | 448 |
| 15 | T0 | 44 | 20 | 87 | 20 | 21 | 30 | 39 | 29 | 29 | 319 |
|  | T1 | 108 | 16 | 86 | 20 | 24 | 30 | 38 | 35 | 29 | 386 |
|  | T2 | 168 | 18 | 89 | 18 | 21 | 28 | 40 | 35 | 29 | 446 |
| 16 | T0 | 59 | 12 | 90 | 12 | 16 | 28 | 27 | 22 | 22 | 288 |
|  | T1 | 108 | 18 | 95 | 18 | 18 | 24 | 38 | 28 | 24 | 371 |
|  | T2 | 58 | 20 | 90 | 24 | 18 | 30 | 34 | 33 | 24 | 331 |
| 17 | T0 | 73 | 19 | 83 | 18 | 18 | 30 | 44 | 33 | 30 | 348 |
|  | T1 | 121.5 | 19 | 92 | 21 | 20 | 30 | 37 | 32 | 30 | 402.5 |
|  | T2 | 113 | 18 | 92 | 24 | 24 | 30 | 37 | 35 | 30 | 403 |
| 18 | T0 | 45 | 17 | 92 | 24 | 24 | 30 | 35 | 35 | 16 | 318 |
|  | T1 | 119.45 | 20 | 95 | 24 | 14 | 29 | 35 | 25 | 30 | 391.45 |
|  | T2 | 111.45 | 20 | 95 | 24 | 22 | 30 | 32 | 28 | 30 | 392.45 |
| 19 | T0 | 203 | 20 | 95 | 24 | 24 | 30 | 36 | 35 | 30 | 497 |
|  | T1 | 160.5 | 20 | 95 | 24 | 24 | 30 | 28 | 35 | 30 | 446.5 |
|  | T2 | 230.5 | 20 | 95 | 24 | 24 | 30 | 33 | 35 | 30 | 521.5 |
| 20 | T0 | 29 | 16 | 87 | 24 | 19 | 28 | 32 | 26 | 27 | 288 |
|  | T1 | 76.5 | 20 | 89 | 12 | 14 | 29 | 46 | 28 | 28 | 342.5 |
|  | T2 | 45 | 15 | 66 | 14 | 16 | 29 | 37 | 31 | 23 | 276 |
| 21 | T0 | 67.5 | 20 | 95 | 24 | 12 | 30 | 46 | 31 | 22 | 347.5 |
|  | T1 | 180.5 | 17 | 90 | 24 | 12 | 30 | 38 | 27 | 30 | 448.5 |
|  | T2 | 158.5 | 18 | 93 | 22 | 14 | 30 | 43 | 27 | 30 | 435.5 |
| 22 | T0 | 29 | 8 | 77 | 18 | 18 | 30 | 33 | 33 | 22 | 268 |
|  | T1 | 101 | 8 | 61 | 15 | 12 | 30 | 27 | 35 | 26 | 315 |
|  | T2 | 108 | 18 | 89 | 24 | 14 | 30 | 34 | 35 | 30 | 382 |
| 23 | T0 | 58 | 16 | 75 | 14 | 18 | 30 | 41 | 35 | 26 | 313 |
|  | T1 | 120.25 | 20 | 86 | 18 | 18 | 30 | 46 | 33 | 27 | 398.25 |
|  | T2 | 101.9 | 17 | 82 | 24 | 24 | 30 | 50 | 35 | 30 | 393.9 |
| 24 | T0 | 45 | 15 | 72 | 17 | 18 | 24 | 37 | 28 | 24 | 280 |
|  | T1 | 140.5 | 16 | 76 | 18 | 18 | 24 | 39 | 28 | 24 | 383.5 |
|  | T2 | 207 | 20 | 95 | 24 | 24 | 30 | 38 | 35 | 30 | 503 |
| 25 | T0 | 47.5 | 15 | 86 | 15 | 11 | 27 | 47 | 31 | 29 | 308.5 |
|  | T1 | 143.5 | 18 | 89 | 18 | 20 | 30 | 32 | 31 | 30 | 411.5 |
|  | T2 | 69.5 | 19 | 83 | 24 | 18 | 24 | 36 | 28 | 24 | 325.5 |
| 26 | T0 | 103.5 | 16 | 76 | 18 | 18 | 24 | 30 | 28 | 24 | 337.5 |
|  | T1 | 177.25 | 20 | 95 | 24 | 24 | 30 | 44 | 35 | 30 | 479.25 |
|  | T2 | 179 | 20 | 95 | 24 | 20 | 30 | 44 | 34 | 30 | 476 |
| 27 | T0 | 95 | 18 | 78 | 15 | 12 | 24 | 31 | 27 | 26 | 326 |
|  | T1 | 152 | 14 | 75 | 21 | 22 | 24 | 35 | 28 | 24 | 395 |
|  | T2 | 159.5 | 18 | 95 | 12 | 12 | 30 | 34 | 35 | 30 | 425.5 |
| 28 | T0 | 58 | 18 | 84 | 12 | 12 | 30 | 36 | 27 | 22 | 299 |
|  | T1 | 112 | 18 | 91 | 23 | 20 | 30 | 36 | 30 | 30 | 390 |
|  | T2 | 106.5 | 19 | 89 | 21 | 18 | 30 | 35 | 31 | 28 | 377.5 |
| 29 | T0 | 126.5 | 20 | 95 | 22 | 24 | 30 | 42 | 35 | 30 | 424.5 |
|  | T1 | 180.5 | 19 | 93 | 24 | 24 | 30 | 38 | 35 | 30 | 473.5 |
|  | T2 | 180.5 | 20 | 95 | 24 | 24 | 30 | 38 | 35 | 30 | 476.5 |
| 30 | T0 | 29 | 10 | 49 | 13 | 8 | 24 | 35 | 25 | 16 | 209 |
|  | T1 | 101 | 12 | 60 | 12 | 11 | 24 | 26 | 26 | 20 | 292 |
|  | T2 | 109 | 12 | 66 | 15 | 14 | 24 | 26 | 27 | 22 | 315 |
| 31 | T0 | 36 | 20 | 95 | 24 | 24 | 30 | 50 | 35 | 30 | 344 |
|  | T1 | 112.5 | 18 | 79 | 18 | 20 | 30 | 34 | 35 | 26 | 372.5 |
|  | T2 | 118 | 18 | 79 | 18 | 18 | 30 | 32 | 35 | 26 | 374 |
| 32 | T0 | 101 | 16 | 79 | 15 | 13 | 30 | 32 | 35 | 30 | 351 |
|  | T1 | 125.95 | 19 | 80 | 15 | 15 | 30 | 33 | 35 | 28 | 380.95 |
|  | T2 | 128 | 19 | 78 | 15 | 15 | 30 | 28 | 33 | 26 | 372 |
| 33 | T0 | 37 | 14 | 68 | 14 | 11 | 24 | 32 | 27 | 21 | 248 |
|  | T1 | 93.5 | 16 | 76 | 17 | 16 | 24 | 37 | 28 | 23 | 330.5 |
|  | T2 | 108 | 18 | 89 | 18 | 15 | 30 | 26 | 31 | 27 | 362 |
| 34 | T0 | 52.45 | 15 | 78 | 15 | 10 | 26 | 41 | 28 | 21 | 286.45 |
|  | T1 | 89.75 | 14 | 74 | 13 | 13 | 30 | 37 | 29 | 25 | 324.75 |
|  | T2 | 101.45 | 18 | 78 | 17 | 15 | 29 | 31 | 32 | 26 | 347.45 |
| 35 | T0 | 82.5 | 16 | 77 | 13 | 12 | 23 | 40 | 30 | 21 | 314.5 |
|  | T1 | 159 | 17 | 75 | 16 | 13 | 23 | 44 | 31 | 26 | 404 |
|  | T2 | 75 | 16 | 77 | 10 | 9 | 21 | 39 | 27 | 22 | 296 |
| 36 | T0 | 58 | 14 | 66 | 12 | 20 | 24 | 31 | 27 | 20 | 272 |
|  | T1 | 58 | 16 | 75 | 12 | 8 | 30 | 33 | 35 | 22 | 289 |
|  | T2 | 58 | 16 | 77 | 15 | 15 | 30 | 30 | 33 | 28 | 302 |
| 37 | T0 | 72 | 18 | 77 | 24 | 24 | 30 | 37 | 35 | 30 | 347 |
|  | T1 | 139.5 | 18 | 84 | 24 | 24 | 30 | 35 | 35 | 30 | 419.5 |
|  | T2 | 125.5 | 16 | 81 | 18 | 18 | 30 | 32 | 33 | 26 | 379.5 |
| 38 | T0 | 72 | 20 | 84 | 13 | 17 | 25 | 43 | 31 | 26 | 331 |
|  | T1 | 118.75 | 16 | 67 | 17 | 16 | 30 | 41 | 35 | 26 | 366.75 |
|  | T2 | 127.5 | 16 | 68 | 16 | 12 | 30 | 36 | 35 | 26 | 366.5 |
| 39 | T0 | 60.45 | 19 | 89 | 18 | 14 | 30 | 46 | 31 | 30 | 337.45 |
|  | T1 | 144.5 | 18 | 93 | 24 | 24 | 29 | 48 | 35 | 30 | 445.5 |
|  | T2 | 146.45 | 20 | 95 | 18 | 14 | 30 | 48 | 33 | 30 | 434.45 |
| 40 | T0 | 44 | 20 | 95 | 23 | 24 | 24 | 46 | 35 | 30 | 341 |
|  | T1 | 49 | 20 | 92 | 24 | 24 | 30 | 42 | 35 | 30 | 346 |
|  | T2 | 89 | 20 | 95 | 24 | 24 | 30 | 50 | 35 | 30 | 397 |
| 41 | T0 | 29 | 11 | 58 | 15 | 11 | 22 | 35 | 20 | 19 | 220 |
|  | T1 | 80 | 15 | 67 | 18 | 9 | 27 | 35 | 22 | 27 | 300 |
|  | T2 | 34 | 15 | 73 | 18 | 12 | 26 | 40 | 24 | 21 | 263 |
| 42 | T0 | 87 | 15 | 62 | 15 | 14 | 24 | 38 | 28 | 24 | 307 |
|  | T1 | 176 | 20 | 95 | 22 | 18 | 30 | 37 | 28 | 26 | 452 |
|  | T2 | 144.5 | 18 | 93 | 24 | 24 | 30 | 50 | 35 | 30 | 448.5 |
| 43 | T0 | 58 | 20 | 95 | 24 | 24 | 30 | 42 | 35 | 28 | 356 |
|  | T1 | 83 | 20 | 95 | 24 | 22 | 29 | 42 | 35 | 23 | 373 |
|  | T2 | 108.5 | 20 | 95 | 24 | 18 | 30 | 42 | 32 | 30 | 399.5 |
| 44 | T0 | 58 | 20 | 95 | 24 | 24 | 30 | 34 | 35 | 30 | 350 |
|  | T1 | 158 | 20 | 95 | 24 | 24 | 30 | 50 | 35 | 30 | 466 |
|  | T2 | 159.5 | 20 | 95 | 24 | 24 | 30 | 50 | 35 | 30 | 467.5 |

**Supplementary table 6.** Raw data of dental plaque index from tester A

| Serial number | Phase | Tooth bit | | | | | | | | | | | | | | | | | | | | total | average |
| --- | --- | --- | --- | --- | --- | --- | --- | --- | --- | --- | --- | --- | --- | --- | --- | --- | --- | --- | --- | --- | --- | --- | --- |
|  |  | 15 | 14 | 13 | 12 | 11 | 21 | 22 | 23 | 24 | 25 | 45 | 44 | 43 | 42 | 41 | 31 | 32 | 33 | 34 | 35 |  |  |
| 1 | T0 | 2 | / | 3 | 4 | 0 | 0 | 0 | 1 | / | 0 | 1 | / | 1 | 3 | 0 | 2 | 0 | 1 | / | 1 | 19 | 1.1875 |
|  | T1 | 3 | / | 2 | 2 | 0 | 0 | 2 | 3 | / | 2 | 1 | / | 2 | 4 | 3 | 3 | 3 | 1 | / | 0 | 31 | 1.9375 |
|  | T2 | 2 | / | 2 | 1 | 0 | 0 | 2 | 1 | / | 2 | 0 | / | 3 | 4 | 2 | 2 | 2 | 2 | / | 1 | 26 | 1.625 |
| 2 | T0 | 1 | / | 1 | 1 | 0 | 0 | 0 | 0 | / | 0 | / | 0 | 1 | 1 | 1 | 1 | 0 | 0 | 0 | / | 7 | 0.4375 |
|  | T1 | 1 | / | 1 | 1 | 1 | 0 | 2 | 1 | / | 1 | / | 0 | 0 | 1 | 1 | 2 | 1 | 0 | 1 | / | 14 | 0.875 |
|  | T2 | 1 | / | 1 | 1 | 0 | 0 | 0 | 1 | / | 1 | / | 0 | 1 | 1 | 1 | 1 | 2 | 1 | 0 | / | 12 | 0.75 |
| 3 | T0 | 3 | / | 4 | 3 | 1 | 1 | 2 | 3 | / | 0 | / | 1 | 1 | / | 0 | 0 | / | 0 | 0 | 1 | 20 | 1.333333 |
|  | T1 | 1 | / | 2 | 1 | 0 | 0 | 3 | 2 | / | 1 | / | 1 | 1 | / | 0 | 1 | / | 1 | 0 | 0 | 14 | 0.933333 |
|  | T2 | 2 | / | 3 | 3 | 1 | 0 | 3 | 3 | / | 2 | / | 3 | 0 | / | 0 | 1 | / | 2 | 2 | 2 | 27 | 1.8 |
| 4 | T0 | 0 | / | 0 | 0 | 0 | 0 | 0 | 0 | / | 0 | 0 | / | 0 | 1 | 0 | 0 | 0 | 0 | 0 | / | 1 | 0.0625 |
|  | T1 | 0 | / | 0 | 1 | 0 | 1 | 2 | 0 | / | 0 | 0 | / | 0 | 1 | 1 | 0 | 0 | 0 | 0 | / | 6 | 0.375 |
|  | T2 | 0 | / | 1 | 0 | 0 | 1 | 2 | 1 | / | 1 | 1 | / | 1 | 1 | 0 | 0 | 1 | 0 | 1 | / | 11 | 0.6875 |
| 5 | T0 | 0 | / | 0 | 0 | 0 | 0 | 0 | 0 | / | 0 | 0 | / | 0 | 0 | 0 | 0 | 0 | 0 | / | 1 | 1 | 0.0625 |
|  | T1 | 2 | / | 1 | 1 | 0 | 0 | 0 | 2 | / | 2 | 2 | / | 2 | 1 | 0 | 0 | 0 | 0 | / | 3 | 16 | 1 |
|  | T2 | 2 | / | 2 | 1 | 0 | 0 | 0 | 0 | / | 2 | 2 | / | 1 | 0 | 0 | 0 | 0 | 0 | / | 2 | 12 | 0.75 |
| 6 | T0 | 0 | / | 0 | 0 | 0 | 0 | 0 | 0 | / | 1 | / | 3 | 2 | 0 | 0 | 1 | 0 | 3 | 2 | / | 12 | 0.75 |
|  | T1 | 1 | / | 0 | 0 | 0 | 0 | 0 | 0 | / | 2 | / | 3 | 3 | 1 | 1 | 1 | 0 | 0 | 1 | / | 13 | 0.8125 |
|  | T2 | 1 | / | 1 | 0 | 0 | 0 | 1 | 1 | / | 2 | / | 2 | 1 | 1 | 2 | 2 | 1 | 1 | 2 | / | 18 | 1.125 |
| 7 | T0 | / | 0 | 1 | 0 | 1 | 0 | 0 | 0 | 1 | / | / | 0 | 1 | 0 | 0 | 0 | 0 | 1 | 0 | / | 5 | 0.3125 |
|  | T1 | / | 1 | 1 | 1 | 1 | 1 | 2 | 1 | 0 | / | / | 2 | 2 | 2 | 1 | 1 | 2 | 2 | 2 | / | 22 | 1.375 |
|  | T2 | / | 0 | 0 | 1 | 0 | 0 | 0 | 0 | 1 | / | / | 2 | 1 | 2 | 1 | 1 | 1 | 1 | 0 | / | 11 | 0.6875 |
| 8 | T0 | / | 0 | 0 | 0 | 0 | 0 | 0 | 1 | / | 1 | 1 | 1 | 1 | 0 | 0 | 0 | 0 | 0 | 1 | 1 | 7 | 0.388889 |
|  | T1 | / | 1 | 2 | 0 | 0 | 1 | 1 | 2 | / | 2 | 2 | 2 | 3 | 3 | 3 | 2 | 2 | 2 | 2 | 2 | 30 | 1.764706 |
|  | T2 | / | 2 | 1 | 0 | 1 | 1 | 2 | 3 | / | 1 | 2 | 2 | 3 | 3 | 3 | 3 | 3 | 1 | 2 | 2 | 33 | 1.941176 |
| 9 | T0 | 0 | / | 0 | 0 | 0 | 0 | 0 | 1 | / | 3 | 0 | 0 | 1 | 0 | 0 | 1 | 0 | 1 | 1 | 0 | 8 | 0.444444 |
|  | T1 | 2 | / | 2 | 1 | 1 | 0 | 2 | 1 | / | 3 | 2 | 2 | 2 | 2 | 2 | 1 | 0 | 2 | 1 | 1 | 26 | 1.529412 |
|  | T2 | 1 | / | 2 | 2 | 0 | 0 | 2 | 1 | / | 2 | 2 | 2 | 3 | 3 | 2 | 1 | 1 | 2 | 1 | 1 | 27 | 1.588235 |
| 10 | T0 | / | 1 | 1 | 1 | 0 | 0 | 0 | 0 | 0 | / | 0 | 0 | 0 | 0 | 1 | 1 | 0 | 0 | 0 | 2 | 7 | 0.388889 |
|  | T1 | / | 2 | 1 | 2 | 1 | 0 | 0 | 0 | 0 | / | 0 | 0 | 0 | 0 | 1 | 0 | 0 | 0 | 2 | 0 | 9 | 0.5 |
|  | T2 | / | 1 | 2 | 3 | 1 | 1 | 1 | 1 | 0 | / | 1 | 0 | 2 | 0 | 2 | 2 | 1 | 1 | 2 | 1 | 22 | 1.222222 |
| 11 | T0 | / | 1 | 2 | 4 | 2 | 1 | 1 | 0 | 1 | / | / | 2 | 3 | 4 | 3 | 2 | 1 | 0 | 1 | / | 28 | 1.75 |
|  | T1 | / | 3 | 2 | 4 | 2 | 2 | 2 | 2 | 3 | / | / | 2 | 2 | 3 | 3 | 4 | 4 | 3 | 3 | / | 44 | 2.75 |
|  | T2 | / | 3 | 2 | 3 | 3 | 2 | 2 | 1 | 2 | / | / | 3 | 3 | 3 | 3 | 2 | 4 | 3 | 2 | / | 41 | 2.5625 |
| 12 | T0 | / | 1 | 1 | 0 | 0 | 0 | 0 | 0 | 0 | / | 2 | 1 | 1 | 0 | 0 | 0 | 0 | 0 | 0 | 0 | 6 | 0.333333 |
|  | T1 | / | 3 | 2 | 2 | 0 | 0 | 0 | 2 | 1 | / | 3 | 3 | 3 | 2 | 3 | 0 | 1 | 0 | 0 | 0 | 25 | 1.388889 |
|  | T2 | / | 2 | 1 | 1 | 0 | 0 | 0 | 0 | 1 | / | 2 | 2 | 2 | 1 | 2 | 2 | 1 | 1 | 1 | 1 | 20 | 1.111111 |
| 13 | T0 | 1 | / | 0 | 0 | 0 | 0 | 0 | 0 | / | 0 | 1 | / | 0 | 0 | 1 | 0 | 0 | 0 | / | 0 | 3 | 0.1875 |
|  | T1 | 1 | / | 1 | 1 | 1 | 0 | 1 | 2 | / | 2 | 0 | / | 1 | 1 | 2 | 2 | 2 | 1 | / | 1 | 19 | 1.1875 |
|  | T2 | 0 | / | 1 | 0 | 0 | 0 | 0 | 0 | / | 1 | 1 | / | 1 | 1 | 1 | 1 | 1 | 1 | / | 0 | 9 | 0.5625 |
| 14 | T0 | 1 | 0 | 0 | 2 | 1 | 0 | 0 | 0 | 0 | 0 | 0 | 0 | 0 | 2 | 1 | 0 | 0 | 0 | 0 | 0 | 7 | 0.35 |
|  | T1 | 1 | 0 | 0 | 0 | 1 | 0 | 0 | 0 | 0 | 1 | 0 | 0 | 0 | 2 | 0 | 0 | 0 | 0 | 1 | 0 | 6 | 0.3 |
|  | T2 | 2 | 0 | 0 | 0 | 0 | 0 | 0 | 1 | 1 | 1 | 1 | 1 | 0 | 1 | 0 | 0 | 0 | 0 | 1 | 1 | 10 | 0.5 |
| 15 | T0 | 0 | 0 | 0 | 0 | 1 | 0 | 0 | 0 | 0 | 0 | 0 | 0 | 1 | 0 | 0 | 0 | 0 | 0 | 0 | 0 | 2 | 0.1 |
|  | T1 | 1 | 0 | 2 | 2 | 1 | 0 | 0 | 1 | 1 | 1 | 1 | 1 | 0 | 2 | 2 | 1 | 0 | 1 | 1 | 1 | 19 | 0.95 |
|  | T2 | 0 | 1 | 1 | 1 | 1 | 0 | 0 | 0 | 0 | 1 | 0 | 0 | 0 | 1 | 1 | 0 | 0 | 1 | 1 | 1 | 10 | 0.5 |
| 16 | T0 | 0 | / | 0 | 0 | 0 | 0 | 0 | 0 | / | 0 | 0 | / | 0 | 1 | 1 | 0 | 0 | 1 | / | 0 | 3 | 0.1875 |
|  | T1 | 2 | / | 1 | 2 | 0 | 0 | 1 | 2 | / | 0 | 2 | / | 2 | 2 | 2 | 0 | 2 | 3 | / | 2 | 23 | 1.4375 |
|  | T2 | 2 | / | 2 | 1 | 1 | 1 | 0 | 1 | / | 2 | 0 | / | 2 | 2 | 2 | 2 | 1 | 2 | / | 2 | 23 | 1.4375 |
| 17 | T0 | 0 | 0 | 0 | 0 | 0 | 2 | 0 | 0 | 0 | 0 | 1 | 0 | 1 | 0 | 0 | 0 | 0 | 1 | 1 | 1 | 7 | 0.35 |
|  | T1 | 1 | 1 | 0 | 1 | 1 | 0 | 0 | 0 | 1 | 2 | 2 | 2 | 2 | 2 | 0 | 0 | 0 | 1 | 2 | 1 | 19 | 0.95 |
|  | T2 | 1 | 2 | 2 | 1 | 1 | 1 | 1 | 2 | 1 | 1 | 1 | 2 | 2 | 1 | 2 | 2 | 1 | 2 | 2 | 2 | 30 | 1.5 |
| 18 | T0 | 0 | 0 | 1 | 2 | 0 | 0 | 0 | 1 | 1 | 0 | 0 | 1 | 1 | 0 | 0 | 0 | / | 0 | 2 | 1 | 10 | 0.526316 |
|  | T1 | 2 | 2 | 2 | 2 | 1 | 0 | 0 | 1 | 1 | 2 | 2 | 1 | 0 | 0 | 0 | 0 | / | 0 | 0 | 1 | 17 | 0.894737 |
|  | T2 | 1 | 2 | 2 | 2 | 1 | 0 | 1 | 3 | 2 | 2 | 2 | 2 | 2 | 1 | 2 | 1 | / | 1 | 2 | 2 | 31 | 1.631579 |
| 19 | T0 | 0 | / | 0 | 0 | 0 | 0 | 0 | 0 | / | 1 | 0 | 0 | 0 | 0 | 0 | 0 | 1 | 0 | 0 | 0 | 2 | 0.111111 |
|  | T1 | 0 | / | 0 | 1 | 0 | 0 | 1 | 0 | / | 1 | 1 | 1 | 0 | 2 | 2 | 1 | 2 | 0 | 0 | 0 | 12 | 0.666667 |
|  | T2 | 0 | / | 0 | 0 | 0 | 0 | 1 | 0 | / | 1 | 1 | 1 | 1 | 2 | 2 | 2 | 2 | 1 | 1 | 1 | 16 | 0.888889 |
| 20 | T0 | 1 | 1 | 2 | 1 | 0 | 0 | 0 | 0 | 0 | 0 | 1 | 1 | 1 | 0 | 0 | 0 | 0 | 0 | 1 | 0 | 9 | 0.45 |
|  | T1 | 1 | 1 | 0 | 1 | 0 | 0 | 0 | 0 | 1 | 1 | 2 | 0 | 2 | 2 | 1 | 2 | 1 | 2 | 2 | 2 | 21 | 1.05 |
|  | T2 | 2 | 2 | 2 | 2 | 0 | 0 | 1 | 0 | 0 | 1 | 0 | 2 | 2 | 0 | 1 | 1 | 1 | 1 | 2 | 2 | 22 | 1.1 |
| 21 | T0 | 0 | / | 0 | 0 | 0 | 0 | 1 | 0 | / | 0 | 0 | / | 0 | 0 | 0 | 0 | 2 | 0 | / | 0 | 3 | 0.1875 |
|  | T1 | 1 | / | 0 | 0 | 1 | 0 | 0 | 0 | / | 1 | 2 | / | 1 | 2 | 2 | 2 | 0 | 1 | / | 1 | 14 | 0.875 |
|  | T2 | 1 | / | 1 | 0 | 0 | 0 | 1 | 0 | / | 1 | 1 | / | 1 | 1 | 2 | 2 | 0 | 0 | / | 2 | 13 | 0.8125 |
| 22 | T0 | 1 | 0 | 0 | 0 | 0 | 0 | 0 | 0 | 0 | 0 | 1 | 0 | 1 | 0 | 1 | 0 | 0 | 0 | 0 | 1 | 5 | 0.25 |
|  | T1 | 2 | 2 | 1 | 2 | 1 | 0 | 1 | 1 | 2 | 2 | 2 | 2 | 2 | 3 | 3 | 2 | 2 | 2 | 1 | 2 | 35 | 1.75 |
|  | T2 | 2 | 2 | 1 | 1 | 0 | 0 | 1 | 2 | 1 | 2 | 2 | 1 | 0 | 0 | 0 | 1 | 1 | 1 | 1 | 2 | 21 | 1.05 |
| 23 | T0 | 0 | / | 0 | 0 | 0 | 0 | 1 | 0 | / | 0 | 0 | / | 0 | 0 | 0 | 0 | 0 | 1 | / | 0 | 2 | 0.125 |
|  | T1 | 2 | / | 2 | 1 | 1 | 0 | 2 | 2 | / | 2 | 2 | / | 2 | 2 | 2 | 2 | 1 | 2 | / | 2 | 27 | 1.6875 |
|  | T2 | 1 | / | 2 | 1 | 1 | 0 | 1 | 2 | / | 2 | 1 | / | 1 | 1 | 0 | 1 | 1 | 1 | / | 2 | 18 | 1.125 |
| 24 | T0 | 0 | / | 1 | 2 | 1 | 2 | 2 | 0 | / | 0 | 0 | 0 | 1 | / | 1 | 0 | / | 0 | 2 | / | 12 | 0.8 |
|  | T1 | 2 | / | 3 | 1 | 1 | 1 | 2 | 2 | / | 1 | 2 | 3 | 2 | / | 2 | 2 | / | 2 | 3 | / | 29 | 1.933333 |
|  | T2 | 1 | / | 3 | 2 | 2 | 1 | 2 | 2 | / | 1 | 2 | 3 | 2 | / | 1 | 2 | / | 3 | 2 | / | 29 | 1.933333 |
| 25 | T0 | 2 | / | 2 | 2 | 1 | 0 | 2 | 0 | / | 0 | 1 | 1 | 1 | 0 | 0 | 0 | 0 | 0 | 0 | 0 | 12 | 0.666667 |
|  | T1 | 2 | / | 2 | 2 | 2 | 2 | 2 | 1 | / | 1 | 1 | 1 | 1 | 1 | 1 | 1 | 1 | 0 | 0 | 1 | 22 | 1.222222 |
|  | T2 | 1 | / | 2 | 2 | 2 | 1 | 1 | 1 | / | 0 | 1 | 1 | 1 | 1 | 1 | 1 | 1 | 0 | 0 | 0 | 17 | 0.944444 |
| 26 | T0 | 1 | 1 | 1 | 0 | 0 | 0 | 0 | 1 | 0 | 1 | / | 1 | 1 | 0 | 0 | 0 | 0 | 0 | 0 | 0 | 7 | 0.368421 |
|  | T1 | 2 | 2 | 2 | 1 | 1 | 1 | 1 | 1 | 2 | 1 | / | 2 | 2 | 2 | 2 | 1 | 0 | 0 | 0 | 1 | 24 | 1.263158 |
|  | T2 | 2 | 2 | 2 | 1 | 0 | 0 | 1 | 1 | 1 | 2 | / | 1 | 1 | 1 | 1 | 1 | 0 | 0 | 1 | 0 | 18 | 0.947368 |
| 27 | T0 | / | 1 | 1 | 0 | 0 | 0 | 0 | 0 | 1 | / | / | 1 | 1 | 0 | 0 | 0 | 0 | 0 | 0 | / | 5 | 0.3125 |
|  | T1 | / | 1 | 2 | 1 | 0 | 0 | 1 | 1 | 0 | / | / | 1 | 1 | 1 | 1 | 0 | 0 | 0 | 2 | / | 12 | 0.75 |
|  | T2 | / | 1 | 2 | 1 | 0 | 0 | 0 | 2 | 0 | / | / | 1 | 2 | 1 | 0 | 0 | 0 | 2 | 1 | / | 13 | 0.8125 |
| 28 | T0 | 1 | / | 0 | 0 | 0 | 0 | 0 | 0 | / | 1 | / | 0 | 0 | 0 | 0 | 0 | 0 | 0 | 0 | / | 2 | 0.125 |
|  | T1 | 1 | / | 1 | 1 | 0 | 0 | 1 | 1 | / | 1 | / | 0 | 0 | 1 | 1 | 0 | 1 | 1 | 1 | / | 11 | 0.6875 |
|  | T2 | 1 | / | 1 | 1 | 0 | 0 | 1 | 0 | / | 1 | / | 1 | 0 | 2 | 1 | 1 | 1 | 2 | 0 | / | 13 | 0.8125 |
| 29 | T0 | / | 0 | 0 | 0 | 0 | 0 | 0 | 0 | / | 1 | 1 | / | 0 | 0 | 0 | 0 | 0 | 0 | / | 1 | 3 | 0.1875 |
|  | T1 | / | 1 | 0 | 0 | 0 | 1 | 1 | 0 | / | 1 | 1 | / | 0 | 0 | 0 | 2 | 0 | 0 | / | 1 | 8 | 0.5 |
|  | T2 | / | 0 | 0 | 1 | 0 | 0 | 0 | 0 | / | 1 | 1 | / | 0 | 0 | 1 | 1 | 0 | 0 | / | 1 | 6 | 0.375 |
| 30 | T0 | 0 | 0 | 0 | 1 | 1 | 1 | 1 | 0 | 0 | 0 | 0 | 0 | 0 | 1 | 0 | 0 | 1 | 0 | 0 | 0 | 6 | 0.3 |
|  | T1 | 2 | 2 | 2 | 1 | 1 | 1 | 1 | 1 | 2 | 2 | 1 | 1 | 1 | 0 | 1 | 2 | 2 | 1 | 1 | 1 | 26 | 1.3 |
|  | T2 | 1 | 2 | 1 | 0 | 1 | 1 | 1 | 1 | 2 | 2 | 1 | 1 | 1 | 0 | 1 | 1 | 0 | 1 | 1 | 1 | 20 | 1 |
| 31 | T0 | 0 | / | 2 | 2 | 2 | 0 | 0 | 0 | / | 0 | 1 | / | 0 | 2 | 0 | 0 | 0 | 2 | / | 0 | 11 | 0.6875 |
|  | T1 | 2 | / | 1 | 2 | 2 | 1 | 0 | 1 | / | 2 | 3 | / | 2 | 3 | 1 | 2 | 2 | 2 | / | 1 | 27 | 1.6875 |
|  | T2 | 1 | / | 1 | 1 | 0 | 1 | 1 | 1 | / | 2 | 1 | / | 1 | 0 | 1 | 0 | 1 | 0 | / | 1 | 13 | 0.8125 |
| 32 | T0 | 0 | 1 | 0 | 1 | 0 | 0 | 0 | 0 | 0 | 0 | 1 | 2 | 1 | 0 | 0 | 0 | 0 | 0 | 0 | 0 | 6 | 0.3 |
|  | T1 | 0 | 0 | 0 | 1 | 1 | 0 | 1 | 1 | 0 | 0 | 2 | 1 | 1 | 0 | 1 | 0 | 0 | 0 | 0 | 0 | 9 | 0.45 |
|  | T2 | 1 | 0 | 0 | 0 | 0 | 0 | 0 | 0 | 0 | 0 | 1 | 1 | 0 | 0 | 0 | 0 | 0 | 0 | 0 | 0 | 3 | 0.15 |
| 33 | T0 | 0 | 0 | 0 | 1 | 1 | 1 | 0 | 0 | 0 | 0 | 0 | 0 | 1 | 0 | 1 | 0 | 0 | 0 | 0 | 0 | 5 | 0.25 |
|  | T1 | 2 | 2 | 2 | 2 | 1 | 0 | 1 | 1 | 2 | 2 | 2 | 2 | 2 | 3 | 3 | 2 | 1 | 1 | 0 | 2 | 33 | 1.65 |
|  | T2 | 2 | 2 | 1 | 0 | 0 | 0 | 0 | 1 | 2 | 2 | 2 | 1 | 3 | 3 | 2 | 2 | 1 | 0 | 0 | 1 | 25 | 1.25 |
| 34 | T0 | / | 1 | 0 | 0 | 0 | 0 | 0 | 0 | 0 | / | 0 | 0 | 2 | 1 | 0 | 0 | 0 | 0 | 0 | 0 | 4 | 0.222222 |
|  | T1 | / | 1 | 1 | 0 | 0 | 0 | 0 | 0 | 0 | / | 0 | 1 | 1 | 1 | 1 | 1 | 1 | 0 | 0 | 1 | 9 | 0.5 |
|  | T2 | / | 0 | 0 | 0 | 0 | 0 | 1 | 0 | 0 | / | 0 | 0 | 0 | 0 | 0 | 0 | 0 | 0 | 0 | 0 | 1 | 0.055556 |
| 35 | T0 | 1 | / | 0 | 2 | 0 | 0 | 0 | 0 | / | 0 | 2 | / | 0 | 1 | 0 | 0 | 0 | 0 | / | 0 | 6 | 0.375 |
|  | T1 | 1 | / | 0 | 0 | 0 | 0 | 0 | 1 | / | 1 | 1 | / | 0 | 0 | 0 | 0 | 0 | 0 | / | 1 | 5 | 0.3125 |
|  | T2 | 1 | / | 1 | 2 | 1 | 0 | 0 | 1 | / | 1 | 2 | / | 2 | 2 | 1 | 1 | 1 | 1 | / | 0 | 17 | 1.0625 |
| 36 | T0 | 0 | 0 | 1 | 1 | 2 | 2 | 0 | 1 | 1 | 1 | 0 | 0 | 2 | 3 | 1 | 0 | 2 | 2 | 1 | 2 | 22 | 1.1 |
|  | T1 | 1 | 2 | 2 | 1 | 2 | 2 | 2 | 3 | 2 | 2 | 2 | 2 | 2 | 3 | 2 | 3 | 3 | 2 | 2 | 1 | 41 | 2.05 |
|  | T2 | 1 | 1 | 2 | 2 | 2 | 2 | 2 | 2 | 1 | 1 | 2 | 2 | 3 | 4 | 2 | 2 | 4 | 3 | 2 | 2 | 42 | 2.1 |
| 37 | T0 | 0 | / | 0 | 0 | 1 | 0 | 0 | 1 | / | 0 | 1 | 1 | 0 | 0 | 0 | 1 | 0 | 1 | 0 | 1 | 7 | 0.388889 |
|  | T1 | 0 | / | 0 | 2 | 2 | 0 | 0 | 0 | / | 0 | 3 | 3 | 3 | 3 | 3 | 2 | 1 | 2 | 2 | 3 | 29 | 1.611111 |
|  | T2 | 1 | / | 2 | 1 | 0 | 0 | 0 | 2 | / | 0 | 2 | 2 | 2 | 3 | 3 | 2 | 2 | 3 | 3 | 3 | 31 | 1.722222 |
| 38 | T0 | 0 | 0 | 0 | 0 | 0 | 0 | 0 | 1 | 0 | 1 | 0 | 0 | 0 | 0 | 0 | 0 | 0 | 0 | 0 | 1 | 3 | 0.15 |
|  | T1 | 1 | 1 | 1 | 0 | 0 | 0 | 0 | 1 | 1 | 2 | 0 | 0 | 1 | 0 | 0 | 0 | 0 | 0 | 0 | 1 | 9 | 0.45 |
|  | T2 | 1 | 1 | 0 | 0 | 0 | 0 | 0 | 0 | 0 | 1 | 0 | 0 | 0 | 0 | 0 | 0 | 0 | 0 | 0 | 0 | 3 | 0.15 |
| 39 | T0 | 0 | 0 | 1 | 1 | 0 | 0 | 0 | 0 | 0 | 0 | 0 | 0 | 1 | 2 | 0 | 0 | 0 | 0 | 0 | 0 | 5 | 0.25 |
|  | T1 | 1 | 0 | 1 | 0 | 0 | 0 | 0 | 0 | 0 | 1 | 1 | 0 | 0 | 0 | 0 | 0 | 0 | 0 | 0 | 0 | 4 | 0.2 |
|  | T2 | 1 | 1 | 0 | 0 | 0 | 0 | 1 | 0 | 0 | 0 | 1 | 0 | 0 | 0 | 0 | 1 | 1 | 0 | 0 | 1 | 7 | 0.35 |
| 40 | T0 | 1 | 1 | 2 | 3 | 2 | 2 | 3 | 3 | 3 | 1 | 0 | 1 | 1 | 2 | 1 | 0 | 0 | 1 | 2 | 1 | 30 | 1.5 |
|  | T1 | 0 | 2 | 3 | 3 | 1 | 2 | 3 | 3 | 3 | 0 | 1 | 1 | 1 | 2 | 1 | 0 | 0 | 2 | 3 | 2 | 33 | 1.65 |
|  | T2 | 2 | 2 | 2 | 1 | 0 | 1 | 3 | 2 | 3 | 2 | 1 | 1 | 0 | 0 | 2 | 0 | 0 | 2 | 3 | 2 | 29 | 1.45 |
| 41 | T0 | 0 | 1 | 0 | 1 | 0 | 1 | 1 | 0 | 0 | 0 | 1 | 0 | 0 | 1 | 0 | 0 | 0 | 1 | 0 | 0 | 7 | 0.35 |
|  | T1 | 1 | 1 | 1 | 1 | 0 | 0 | 2 | 0 | 1 | 1 | 2 | 2 | 2 | 2 | 2 | 1 | 1 | 1 | 2 | 2 | 25 | 1.25 |
|  | T2 | 2 | 1 | 1 | 2 | 1 | 0 | 2 | 0 | 1 | 2 | 2 | 1 | 2 | 3 | 3 | 2 | 0 | 3 | 2 | 2 | 32 | 1.6 |
| 42 | T0 | 0 | / | 0 | 0 | 0 | 0 | 0 | 0 | / | 0 | 0 | / | 1 | 0 | 0 | 0 | 0 | 0 | / | 1 | 2 | 0.125 |
|  | T1 | 3 | / | 3 | 3 | 1 | 1 | 1 | 2 | / | 2 | 3 | / | 3 | 3 | 2 | 2 | 2 | 3 | / | 2 | 36 | 2.25 |
|  | T2 | 2 | / | 2 | 2 | 1 | 1 | 1 | 2 | / | 2 | 1 | / | 3 | 2 | 2 | 2 | 2 | 2 | / | 2 | 29 | 1.8125 |
| 43 | T0 | / | 1 | 1 | 0 | 0 | 0 | 1 | 0 | 0 | / | 1 | 0 | 0 | 1 | 0 | 0 | 0 | 0 | 0 | 1 | 6 | 0.333333 |
|  | T1 | / | 4 | 3 | 4 | 0 | 0 | 1 | 2 | 3 | / | 3 | 3 | 3 | 3 | 3 | 2 | 3 | 2 | 2 | 2 | 43 | 2.388889 |
|  | T2 | / | 3 | 3 | 4 | 1 | 0 | 1 | 2 | 1 | / | 2 | 2 | 3 | 3 | 3 | 2 | 2 | 1 | 1 | 1 | 35 | 1.944444 |
| 44 | T0 | / | 0 | 1 | 0 | 0 | 0 | 1 | 1 | 1 | / | 0 | 0 | 0 | 0 | 1 | / | 0 | 1 | 2 | 1 | 9 | 0.529412 |
|  | T1 | / | 2 | 3 | 2 | 1 | 0 | 3 | 3 | 2 | / | 2 | 2 | 2 | 1 | 1 | / | 1 | 2 | 3 | 3 | 33 | 1.941176 |
|  | T2 | / | 1 | 1 | 1 | 1 | 1 | 2 | 2 | 1 | / | 2 | 2 | 0 | 0 | 1 | / | 1 | 2 | 1 | 1 | 20 | 1.176471 |

/: the missing tooth

**Supplementary table 7.** Raw data of dental plaque index from tester B

| Serial number | Phase | Tooth bit | | | | | | | | | | | | | | | | | | | | total | average |
| --- | --- | --- | --- | --- | --- | --- | --- | --- | --- | --- | --- | --- | --- | --- | --- | --- | --- | --- | --- | --- | --- | --- | --- |
|  |  | 15 | 14 | 13 | 12 | 11 | 21 | 22 | 23 | 24 | 25 | 45 | 44 | 43 | 42 | 41 | 31 | 32 | 33 | 34 | 35 |  |  |
| 1 | T0 | 2 | / | 3 | 4 | 0 | 0 | 2 | 3 | / | 4 | 1 | / | 1 | 4 | 2 | 2 | 3 | 3 | / | 3 | 37 | 2.3125 |
|  | T1 | 3 | / | 2 | 2 | 0 | 0 | 2 | 3 | / | 2 | 3 | / | 2 | 4 | 1 | 1 | 1 | 3 | / | 2 | 31 | 1.9375 |
|  | T2 | 2 | / | 1 | 0 | 0 | 0 | 0 | 1 | / | 0 | 0 | / | 0 | 2 | 1 | 1 | 0 | 0 | / | 0 | 8 | 0.5 |
| 2 | T0 | 1 | / | 1 | 0 | 0 | 0 | 1 | 0 | / | 0 | / | 0 | 0 | 0 | 1 | 1 | 0 | 0 | 0 | / | 5 | 0.3125 |
|  | T1 | 1 | / | 1 | 0 | 1 | 0 | 0 | 1 | / | 1 | / | 0 | 1 | 1 | 0 | 0 | 1 | 0 | 0 | / | 8 | 0.5 |
|  | T2 | 1 | / | 1 | 0 | 0 | 0 | 0 | 0 | / | 1 | / | 0 | 1 | 0 | 0 | 1 | 0 | 1 | 0 | / | 6 | 0.375 |
| 3 | T0 | 3 | / | 2 | 3 | 0 | 0 | 2 | 1 | / | 0 | / | 2 | 1 | / | 0 | 0 | / | 0 | 0 | 0 | 14 | 0.933333 |
|  | T1 | 1 | / | 2 | 1 | 0 | 0 | 1 | 2 | / | 1 | / | 1 | 1 | / | 0 | 0 | / | 1 | 2 | 1 | 14 | 0.933333 |
|  | T2 | 2 | / | 3 | 3 | 1 | 2 | 1 | 1 | / | 0 | / | 1 | 2 | / | 2 | 1 | / | 0 | 0 | 0 | 19 | 1.266667 |
| 4 | T0 | 0 | / | 0 | 0 | 0 | 0 | 2 | 0 | / | 0 | 0 | / | 0 | 1 | 0 | 0 | 2 | 0 | 0 | / | 5 | 0.3125 |
|  | T1 | 0 | / | 1 | 1 | 0 | 0 | 2 | 2 | / | 0 | 0 | / | 0 | 1 | 0 | 0 | 0 | 0 | 1 | / | 8 | 0.5 |
|  | T2 | 2 | / | 1 | 0 | 0 | 1 | 2 | 0 | / | 1 | 0 | / | 1 | 0 | 0 | 0 | 0 | 0 | 1 | / | 9 | 0.5625 |
| 5 | T0 | 0 | / | 0 | 0 | 0 | 0 | 0 | 0 | / | 0 | 0 | / | 0 | 0 | 0 | 0 | 0 | 0 | / | 1 | 1 | 0.0625 |
|  | T1 | 2 | / | 1 | 1 | 0 | 0 | 0 | 2 | / | 2 | 2 | / | 2 | 1 | 0 | 0 | 0 | 2 | / | 1 | 16 | 1 |
|  | T2 | 2 | / | 0 | 1 | 0 | 0 | 2 | 0 | / | 2 | 2 | / | 1 | 2 | 0 | 0 | 0 | 2 | / | 2 | 16 | 1 |
| 6 | T0 | 0 | / | 0 | 0 | 0 | 0 | 0 | 0 | / | 1 | / | 3 | 4 | 0 | 0 | 0 | 0 | 3 | 3 | / | 14 | 0.875 |
|  | T1 | 1 | / | 1 | 1 | 0 | 0 | 0 | 0 | / | 0 | / | 1 | 1 | 1 | 1 | 1 | 0 | 2 | 1 | / | 11 | 0.6875 |
|  | T2 | 1 | / | 0 | 0 | 0 | 0 | 1 | 1 | / | 2 | / | 2 | 1 | 1 | 2 | 2 | 0 | 1 | 2 | / | 16 | 1 |
| 7 | T0 | / | 0 | 1 | 0 | 1 | 0 | 0 | 0 | 1 | / | / | 0 | 1 | 0 | 0 | 2 | 0 | 1 | 0 | / | 7 | 0.4375 |
|  | T1 | / | 1 | 1 | 1 | 0 | 1 | 2 | 1 | 4 | / | / | 2 | 1 | 2 | 0 | 0 | 0 | 2 | 2 | / | 20 | 1.25 |
|  | T2 | / | 0 | 0 | 1 | 0 | 0 | 0 | 0 | 1 | / | / | 2 | 1 | 2 | 1 | 1 | 1 | 1 | 2 | / | 13 | 0.8125 |
| 8 | T0 | / | 0 | 0 | 0 | 0 | 0 | 0 | 1 | / | 1 | 1 | 0 | 1 | 0 | 0 | 0 | 0 | 0 | 0 | 1 | 5 | 0.277778 |
|  | T1 | / | 3 | 2 | 2 | 0 | 1 | 2 | 4 | / | 2 | 2 | 2 | 1 | 3 | 2 | 2 | 2 | 0 | 2 | 0 | 32 | 1.777778 |
|  | T2 | / | 1 | 1 | 2 | 1 | 1 | 2 | 3 | / | 1 | 2 | 2 | 3 | 3 | 3 | 3 | 3 | 1 | 3 | 0 | 35 | 1.944444 |
| 9 | T0 | 2 | / | 2 | 0 | 0 | 0 | 0 | 1 | / | 1 | 0 | 0 | 1 | 0 | 0 | 0 | 0 | 1 | 0 | 0 | 8 | 0.444444 |
|  | T1 | 2 | / | 2 | 1 | 1 | 0 | 2 | 1 | / | 3 | 2 | 2 | 2 | 2 | 0 | 2 | 2 | 0 | 2 | 1 | 27 | 1.5 |
|  | T2 | 1 | / | 1 | 2 | 0 | 0 | 2 | 1 | / | 2 | 2 | 2 | 3 | 3 | 2 | 1 | 1 | 2 | 2 | 1 | 28 | 1.555556 |
| 10 | T0 | / | 1 | 1 | 1 | 2 | 0 | 0 | 0 | 0 | / | 0 | 0 | 0 | 1 | 1 | 0 | 0 | 0 | 0 | 0 | 7 | 0.388889 |
|  | T1 | / | 2 | 1 | 1 | 0 | 0 | 1 | 2 | 0 | / | 0 | 0 | 0 | 0 | 0 | 0 | 0 | 0 | 0 | 0 | 7 | 0.388889 |
|  | T2 | / | 0 | 2 | 1 | 1 | 1 | 1 | 1 | 0 | / | 1 | 0 | 2 | 4 | 2 | 2 | 3 | 1 | 2 | 0 | 24 | 1.333333 |
| 11 | T0 | / | 3 | 4 | 4 | 4 | 1 | 0 | 2 | 0 | / | / | 0 | 1 | 2 | 2 | 2 | 0 | 0 | 1 | / | 26 | 1.625 |
|  | T1 | / | 3 | 4 | 4 | 4 | 2 | 2 | 2 | 3 | / | / | 2 | 2 | 4 | 4 | 4 | 4 | 3 | 2 | / | 49 | 3.0625 |
|  | T2 | / | 3 | 2 | 3 | 4 | 2 | 2 | 3 | 2 | / | / | 3 | 3 | 3 | 4 | 4 | 3 | 2 | 2 | / | 45 | 2.8125 |
| 12 | T0 | / | 3 | 3 | 2 | 0 | 0 | 0 | 0 | 0 | / | 0 | 1 | 1 | 0 | 0 | 0 | 0 | 0 | 0 | 0 | 10 | 0.555556 |
|  | T1 | / | 3 | 3 | 2 | 0 | 0 | 0 | 0 | 2 | / | 3 | 3 | 4 | 2 | 4 | 2 | 1 | 0 | 0 | 0 | 29 | 1.611111 |
|  | T2 | / | 2 | 1 | 1 | 0 | 0 | 2 | 0 | 1 | / | 3 | 2 | 2 | 1 | 4 | 2 | 1 | 1 | 1 | 1 | 25 | 1.388889 |
| 13 | T0 | 1 | / | 0 | 0 | 0 | 0 | 0 | 0 | / | 0 | 1 | / | 0 | 2 | 1 | 0 | 0 | 0 | / | 0 | 5 | 0.3125 |
|  | T1 | 1 | / | 1 | 1 | 1 | 0 | 1 | 2 | / | 0 | 0 | / | 1 | 1 | 2 | 2 | 2 | 3 | / | 1 | 19 | 1.1875 |
|  | T2 | 2 | / | 1 | 0 | 0 | 0 | 0 | 2 | / | 1 | 1 | / | 0 | 1 | 1 | 1 | 0 | 1 | / | 0 | 11 | 0.6875 |
| 14 | T0 | 1 | 0 | 2 | 2 | 0 | 0 | 0 | 0 | 0 | 0 | 0 | 0 | 0 | 1 | 1 | 0 | 0 | 0 | 0 | 0 | 7 | 0.35 |
|  | T1 | 1 | 0 | 0 | 0 | 1 | 0 | 0 | 0 | 2 | 1 | 0 | 0 | 0 | 0 | 0 | 0 | 0 | 0 | 1 | 0 | 6 | 0.3 |
|  | T2 | 2 | 0 | 0 | 0 | 0 | 0 | 0 | 0 | 1 | 2 | 1 | 1 | 0 | 1 | 0 | 0 | 0 | 0 | 1 | 1 | 10 | 0.5 |
| 15 | T0 | 0 | 0 | 0 | 0 | 1 | 0 | 0 | 0 | 0 | 0 | 0 | 0 | 1 | 0 | 0 | 0 | 0 | 0 | 0 | 2 | 4 | 0.2 |
|  | T1 | 1 | 2 | 2 | 2 | 1 | 0 | 0 | 1 | 1 | 1 | 0 | 1 | 2 | 1 | 1 | 1 | 0 | 1 | 0 | 1 | 19 | 0.95 |
|  | T2 | 0 | 0 | 1 | 1 | 1 | 0 | 0 | 2 | 0 | 1 | 0 | 0 | 2 | 1 | 1 | 0 | 0 | 1 | 1 | 0 | 12 | 0.6 |
| 16 | T0 | 0 | / | 0 | 1 | 0 | 0 | 0 | 0 | / | 0 | 0 | / | 0 | 1 | 0 | 0 | 0 | 1 | / | 0 | 3 | 0.1875 |
|  | T1 | 2 | / | 1 | 0 | 0 | 0 | 1 | 2 | / | 2 | 2 | / | 3 | 3 | 2 | 2 | 2 | 3 | / | 2 | 27 | 1.6875 |
|  | T2 | 2 | / | 2 | 1 | 1 | 1 | 0 | 1 | / | 2 | 2 | / | 2 | 2 | 2 | 2 | 1 | 2 | / | 2 | 25 | 1.5625 |
| 17 | T0 | 0 | 0 | 0 | 2 | 0 | 0 | 2 | 0 | 0 | 0 | 1 | 0 | 1 | 0 | 0 | 0 | 0 | 1 | 1 | 1 | 9 | 0.45 |
|  | T1 | 1 | 0 | 0 | 1 | 1 | 0 | 2 | 1 | 1 | 0 | 2 | 2 | 0 | 0 | 0 | 0 | 0 | 1 | 2 | 1 | 15 | 0.75 |
|  | T2 | 1 | 2 | 2 | 1 | 1 | 1 | 1 | 2 | 1 | 1 | 1 | 2 | 4 | 1 | 0 | 0 | 1 | 2 | 2 | 2 | 28 | 1.4 |
| 18 | T0 | 0 | 0 | 1 | 2 | 0 | 0 | 0 | 1 | 1 | 0 | 2 | 2 | 0 | 0 | 0 | 0 | / | 2 | 0 | 1 | 12 | 0.631579 |
|  | T1 | 2 | 2 | 0 | 0 | 1 | 0 | 0 | 1 | 3 | 2 | 2 | 1 | 0 | 0 | 0 | 0 | / | 0 | 0 | 3 | 17 | 0.894737 |
|  | T2 | 1 | 2 | 2 | 0 | 1 | 2 | 1 | 3 | 2 | 2 | 2 | 2 | 2 | 1 | 2 | 1 | / | 1 | 2 | 2 | 31 | 1.631579 |
| 19 | T0 | 0 | / | 0 | 0 | 0 | 0 | 0 | 0 | / | 1 | 0 | 0 | 0 | 0 | 2 | 0 | 1 | 2 | 0 | 0 | 6 | 0.333333 |
|  | T1 | 0 | / | 0 | 1 | 0 | 0 | 1 | 0 | / | 1 | 1 | 1 | 0 | 2 | 2 | 1 | 2 | 0 | 0 | 0 | 12 | 0.666667 |
|  | T2 | 0 | / | 0 | 1 | 0 | 0 | 1 | 1 | / | 1 | 1 | 1 | 0 | 2 | 2 | 0 | 2 | 1 | 1 | 1 | 15 | 0.833333 |
| 20 | T0 | 1 | 1 | 2 | 1 | 0 | 0 | 0 | 2 | 1 | 0 | 0 | 1 | 1 | 0 | 0 | 0 | 0 | 0 | 1 | 2 | 13 | 0.65 |
|  | T1 | 3 | 3 | 4 | 3 | 0 | 0 | 0 | 2 | 1 | 1 | 2 | 0 | 2 | 0 | 1 | 0 | 1 | 2 | 2 | 0 | 27 | 1.35 |
|  | T2 | 0 | 2 | 2 | 2 | 0 | 0 | 1 | 2 | 1 | 0 | 0 | 0 | 2 | 2 | 1 | 1 | 1 | 1 | 2 | 0 | 20 | 1 |
| 21 | T0 | 2 | / | 0 | 0 | 0 | 0 | 1 | 0 | / | 0 | 0 | / | 0 | 0 | 0 | 0 | 2 | 0 | / | 0 | 5 | 0.3125 |
|  | T1 | 1 | / | 0 | 0 | 1 | 0 | 0 | 0 | / | 1 | 2 | / | 1 | 2 | 2 | 2 | 2 | 1 | / | 3 | 18 | 1.125 |
|  | T2 | 1 | / | 1 | 0 | 0 | 0 | 1 | 0 | / | 1 | 1 | / | 1 | 1 | 2 | 2 | 2 | 0 | / | 2 | 15 | 0.9375 |
| 22 | T0 | 1 | 0 | 0 | 0 | 0 | 0 | 0 | 0 | 0 | 0 | 0 | 0 | 0 | 1 | 1 | 0 | 0 | 0 | 0 | 2 | 5 | 0.25 |
|  | T1 | 2 | 2 | 1 | 2 | 1 | 0 | 1 | 1 | 0 | 2 | 2 | 2 | 4 | 3 | 3 | 2 | 2 | 0 | 1 | 2 | 33 | 1.65 |
|  | T2 | 2 | 2 | 1 | 1 | 0 | 0 | 1 | 0 | 1 | 2 | 2 | 1 | 2 | 0 | 0 | 1 | 3 | 1 | 1 | 2 | 23 | 1.15 |
| 23 | T0 | 0 | / | 0 | 0 | 0 | 0 | 1 | 0 | / | 0 | 0 | / | 2 | 0 | 0 | 0 | 0 | 1 | / | 0 | 4 | 0.25 |
|  | T1 | 2 | / | 4 | 1 | 1 | 0 | 2 | 0 | / | 4 | 2 | / | 2 | 0 | 0 | 2 | 1 | 2 | / | 2 | 25 | 1.5625 |
|  | T2 | 1 | / | 2 | 1 | 1 | 0 | 1 | 1 | / | 2 | 1 | / | 1 | 1 | 0 | 0 | 1 | 1 | / | 2 | 16 | 1 |
| 24 | T0 | 0 | / | 3 | 2 | 1 | 2 | 0 | 0 | / | 0 | 0 | 0 | 1 | / | 1 | 0 | / | 0 | 0 | / | 10 | 0.666667 |
|  | T1 | 2 | / | 3 | 3 | 1 | 1 | 2 | 2 | / | 1 | 2 | 3 | 2 | / | 2 | 2 | / | 2 | 3 | / | 31 | 2.066667 |
|  | T2 | 1 | / | 4 | 2 | 2 | 0 | 4 | 2 | / | 1 | 2 | 3 | 2 | / | 1 | 2 | / | 3 | 4 | / | 33 | 2.2 |
| 25 | T0 | 2 | / | 2 | 2 | 1 | 2 | 0 | 0 | / | 0 | 1 | 1 | 1 | 0 | 0 | 0 | 0 | 0 | 0 | 0 | 12 | 0.666667 |
|  | T1 | 2 | / | 2 | 2 | 2 | 2 | 2 | 1 | / | 0 | 1 | 1 | 1 | 1 | 1 | 1 | 1 | 0 | 0 | 0 | 20 | 1.111111 |
|  | T2 | 1 | / | 2 | 2 | 1 | 1 | 1 | 0 | / | 0 | 1 | 1 | 1 | 2 | 1 | 1 | 0 | 0 | 0 | 0 | 15 | 0.833333 |
| 26 | T0 | 0 | 1 | 1 | 0 | 0 | 0 | 0 | 0 | 0 | 0 | / | 0 | 1 | 0 | 0 | 0 | 0 | 0 | 0 | 1 | 4 | 0.210526 |
|  | T1 | 2 | 2 | 2 | 1 | 1 | 1 | 1 | 1 | 2 | 3 | / | 0 | 2 | 2 | 2 | 1 | 0 | 0 | 0 | 1 | 24 | 1.263158 |
|  | T2 | 2 | 2 | 2 | 0 | 0 | 0 | 1 | 1 | 2 | 2 | / | 1 | 1 | 1 | 1 | 1 | 0 | 0 | 1 | 0 | 18 | 0.947368 |
| 27 | T0 | / | 0 | 1 | 0 | 0 | 0 | 0 | 0 | 0 | / | / | 1 | 1 | 0 | 0 | 0 | 0 | 0 | 0 | / | 3 | 0.1875 |
|  | T1 | / | 1 | 2 | 1 | 0 | 0 | 1 | 1 | 0 | / | / | 1 | 1 | 1 | 0 | 0 | 0 | 0 | 1 | / | 10 | 0.625 |
|  | T2 | / | 1 | 2 | 1 | 0 | 0 | 0 | 2 | 0 | / | / | 1 | 2 | 1 | 0 | 0 | 0 | 0 | 1 | / | 11 | 0.6875 |
| 28 | T0 | 1 | / | 0 | 0 | 0 | 0 | 0 | 0 | / | 1 | / | 0 | 0 | 0 | 0 | 0 | 0 | 0 | 2 | / | 4 | 0.25 |
|  | T1 | 1 | / | 1 | 1 | 0 | 0 | 1 | 0 | / | 1 | / | 0 | 0 | 0 | 1 | 2 | 0 | 1 | 0 | / | 9 | 0.5625 |
|  | T2 | 1 | / | 1 | 1 | 0 | 0 | 1 | 0 | / | 1 | / | 1 | 0 | 2 | 1 | 1 | 1 | 0 | 0 | / | 11 | 0.6875 |
| 29 | T0 | / | 0 | 0 | 0 | 0 | 0 | 0 | 0 | / | 1 | 1 | / | 0 | 0 | 0 | 0 | 0 | 0 | / | 1 | 3 | 0.1875 |
|  | T1 | / | 1 | 0 | 0 | 0 | 1 | 1 | 0 | / | 3 | 1 | / | 2 | 0 | 0 | 0 | 0 | 0 | / | 1 | 10 | 0.625 |
|  | T2 | / | 0 | 0 | 1 | 2 | 2 | 0 | 0 | / | 1 | 0 | / | 0 | 0 | 1 | 1 | 0 | 0 | / | 0 | 8 | 0.5 |
| 30 | T0 | 0 | 0 | 0 | 1 | 1 | 1 | 1 | 0 | 0 | 0 | 0 | 0 | 0 | 1 | 0 | 0 | 1 | 0 | 0 | 0 | 6 | 0.3 |
|  | T1 | 2 | 2 | 2 | 1 | 1 | 1 | 1 | 3 | 0 | 0 | 1 | 1 | 3 | 2 | 1 | 0 | 0 | 1 | 3 | 1 | 26 | 1.3 |
|  | T2 | 1 | 0 | 1 | 2 | 1 | 1 | 1 | 1 | 2 | 2 | 1 | 1 | 1 | 2 | 0 | 0 | 2 | 1 | 1 | 1 | 22 | 1.1 |
| 31 | T0 | 2 | / | 2 | 2 | 0 | 0 | 0 | 0 | / | 0 | 1 | / | 2 | 2 | 0 | 0 | 0 | 2 | / | 0 | 13 | 0.8125 |
|  | T1 | 2 | / | 1 | 2 | 2 | 2 | 0 | 1 | / | 2 | 4 | / | 2 | 4 | 1 | 2 | 2 | 3 | / | 1 | 31 | 1.9375 |
|  | T2 | 1 | / | 1 | 3 | 0 | 1 | 1 | 1 | / | 2 | 1 | / | 1 | 0 | 1 | 2 | 0 | 0 | / | 1 | 16 | 1 |
| 32 | T0 | 0 | 1 | 0 | 1 | 0 | 0 | 0 | 0 | 0 | 0 | 1 | 0 | 1 | 0 | 0 | 0 | 0 | 0 | 0 | 0 | 4 | 0.2 |
|  | T1 | 0 | 0 | 0 | 1 | 1 | 0 | 1 | 1 | 0 | 0 | 2 | 1 | 1 | 2 | 1 | 0 | 0 | 0 | 0 | 0 | 11 | 0.55 |
|  | T2 | 1 | 0 | 0 | 0 | 0 | 0 | 2 | 0 | 0 | 0 | 0 | 0 | 1 | 0 | 0 | 0 | 0 | 0 | 0 | 1 | 5 | 0.25 |
| 33 | T0 | 0 | 0 | 0 | 1 | 1 | 1 | 0 | 0 | 0 | 0 | 0 | 0 | 0 | 1 | 1 | 0 | 0 | 0 | 0 | 0 | 5 | 0.25 |
|  | T1 | 2 | 2 | 2 | 2 | 1 | 0 | 1 | 1 | 2 | 2 | 2 | 0 | 2 | 3 | 3 | 2 | 1 | 1 | 2 | 2 | 33 | 1.65 |
|  | T2 | 2 | 2 | 1 | 0 | 0 | 0 | 0 | 1 | 2 | 2 | 2 | 1 | 3 | 3 | 2 | 2 | 1 | 0 | 0 | 1 | 25 | 1.25 |
| 34 | T0 | / | 1 | 0 | 0 | 0 | 0 | 0 | 0 | 0 | / | 0 | 0 | 0 | 1 | 0 | 0 | 2 | 0 | 0 | 0 | 4 | 0.222222 |
|  | T1 | / | 1 | 0 | 0 | 0 | 0 | 0 | 0 | 0 | / | 0 | 1 | 1 | 2 | 1 | 0 | 1 | 0 | 0 | 0 | 7 | 0.388889 |
|  | T2 | / | 0 | 0 | 0 | 0 | 0 | 0 | 0 | 0 | / | 0 | 0 | 0 | 0 | 0 | 0 | 0 | 0 | 0 | 0 | 0 | 0 |
| 35 | T0 | 1 | / | 0 | 2 | 0 | 0 | 0 | 0 | / | 2 | 0 | / | 0 | 1 | 0 | 0 | 0 | 0 | / | 0 | 6 | 0.375 |
|  | T1 | 1 | / | 0 | 0 | 0 | 0 | 0 | 0 | / | 1 | 0 | / | 0 | 0 | 0 | 0 | 0 | 0 | / | 1 | 3 | 0.1875 |
|  | T2 | 1 | / | 1 | 2 | 1 | 0 | 0 | 1 | / | 1 | 2 | / | 2 | 2 | 1 | 0 | 1 | 1 | / | 0 | 16 | 1 |
| 36 | T0 | 0 | 0 | 1 | 1 | 2 | 2 | 0 | 1 | 1 | 1 | 0 | 0 | 2 | 3 | 3 | 2 | 2 | 0 | 1 | 2 | 24 | 1.2 |
|  | T1 | 1 | 2 | 2 | 1 | 2 | 2 | 2 | 1 | 2 | 2 | 2 | 2 | 2 | 3 | 2 | 3 | 3 | 2 | 2 | 1 | 39 | 1.95 |
|  | T2 | 1 | 1 | 2 | 2 | 2 | 2 | 2 | 2 | 1 | 1 | 0 | 2 | 3 | 4 | 4 | 4 | 4 | 1 | 2 | 2 | 42 | 2.1 |
| 37 | T0 | 0 | / | 0 | 0 | 1 | 0 | 0 | 1 | / | 0 | 1 | 1 | 0 | 0 | 0 | 1 | 0 | 1 | 0 | 1 | 7 | 0.388889 |
|  | T1 | 0 | / | 2 | 2 | 2 | 2 | 2 | 2 | / | 0 | 3 | 3 | 3 | 3 | 1 | 2 | 1 | 2 | 2 | 3 | 35 | 1.944444 |
|  | T2 | 1 | / | 2 | 1 | 0 | 0 | 0 | 2 | / | 0 | 2 | 2 | 2 | 3 | 3 | 2 | 2 | 1 | 1 | 1 | 25 | 1.388889 |
| 38 | T0 | 0 | 0 | 0 | 0 | 0 | 0 | 0 | 0 | 0 | 1 | 0 | 0 | 0 | 0 | 0 | 0 | 0 | 0 | 0 | 1 | 2 | 0.1 |
|  | T1 | 1 | 1 | 0 | 0 | 0 | 0 | 1 | 1 | 1 | 2 | 0 | 0 | 1 | 0 | 0 | 0 | 0 | 0 | 0 | 1 | 9 | 0.45 |
|  | T2 | 1 | 1 | 0 | 0 | 0 | 0 | 0 | 0 | 0 | 1 | 0 | 0 | 0 | 2 | 0 | 0 | 0 | 0 | 0 | 0 | 5 | 0.25 |
| 39 | T0 | 0 | 0 | 0 | 0 | 2 | 0 | 0 | 0 | 0 | 0 | 0 | 0 | 0 | 2 | 1 | 0 | 0 | 0 | 0 | 0 | 5 | 0.25 |
|  | T1 | 1 | 0 | 0 | 0 | 0 | 0 | 0 | 0 | 0 | 1 | 1 | 0 | 0 | 0 | 0 | 0 | 0 | 1 | 0 | 0 | 4 | 0.2 |
|  | T2 | 1 | 1 | 0 | 0 | 0 | 0 | 1 | 0 | 0 | 0 | 1 | 0 | 0 | 0 | 0 | 1 | 1 | 0 | 0 | 1 | 7 | 0.35 |
| 40 | T0 | 1 | 1 | 2 | 3 | 2 | 2 | 3 | 1 | 1 | 4 | 3 | 1 | 1 | 0 | 1 | 0 | 0 | 1 | 2 | 1 | 30 | 1.5 |
|  | T1 | 4 | 2 | 1 | 3 | 1 | 2 | 3 | 1 | 1 | 2 | 3 | 3 | 1 | 0 | 1 | 2 | 0 | 0 | 1 | 2 | 33 | 1.65 |
|  | T2 | 2 | 3 | 3 | 3 | 2 | 0 | 1 | 2 | 3 | 2 | 3 | 3 | 1 | 2 | 0 | 0 | 0 | 0 | 1 | 2 | 33 | 1.65 |
| 41 | T0 | 0 | 1 | 0 | 1 | 0 | 1 | 1 | 0 | 0 | 2 | 1 | 0 | 0 | 1 | 0 | 0 | 0 | 1 | 0 | 0 | 9 | 0.45 |
|  | T1 | 1 | 1 | 0 | 0 | 0 | 0 | 2 | 0 | 1 | 1 | 2 | 0 | 2 | 2 | 2 | 1 | 1 | 1 | 2 | 2 | 21 | 1.05 |
|  | T2 | 2 | 1 | 1 | 2 | 1 | 2 | 2 | 0 | 3 | 0 | 2 | 3 | 2 | 3 | 1 | 2 | 2 | 1 | 2 | 2 | 34 | 1.7 |
| 42 | T0 | 0 | / | 0 | 2 | 0 | 0 | 0 | 0 | / | 0 | 0 | / | 1 | 0 | 0 | 0 | 0 | 0 | / | 1 | 4 | 0.25 |
|  | T1 | 3 | / | 4 | 3 | 2 | 1 | 2 | 2 | / | 2 | 3 | / | 4 | 3 | 2 | 2 | 2 | 3 | / | 2 | 40 | 2.5 |
|  | T2 | 0 | / | 4 | 2 | 3 | 3 | 3 | 0 | / | 0 | 1 | / | 3 | 2 | 0 | 2 | 2 | 2 | / | 0 | 27 | 1.6875 |
| 43 | T0 | / | 1 | 1 | 0 | 0 | 0 | 1 | 0 | 0 | / | 1 | 0 | 0 | 1 | 0 | 0 | 0 | 0 | 0 | 1 | 6 | 0.333333 |
|  | T1 | / | 4 | 4 | 4 | 3 | 0 | 1 | 2 | 3 | / | 3 | 1 | 4 | 4 | 1 | 2 | 1 | 2 | 3 | 3 | 45 | 2.5 |
|  | T2 | / | 3 | 3 | 4 | 3 | 2 | 1 | 0 | 1 | / | 2 | 1 | 3 | 4 | 1 | 0 | 0 | 1 | 3 | 3 | 35 | 1.944444 |
| 44 | T0 | / | 0 | 1 | 0 | 0 | 0 | 1 | 1 | 1 | / | 0 | 0 | 0 | 0 | 1 | / | 0 | 1 | 2 | 1 | 9 | 0.529412 |
|  | T1 | / | 2 | 3 | 2 | 1 | 0 | 3 | 3 | 2 | / | 2 | 2 | 2 | 1 | 1 | / | 1 | 2 | 3 | 1 | 31 | 1.823529 |
|  | T2 | / | 1 | 1 | 1 | 1 | 1 | 2 | 2 | 1 | / | 2 | 2 | 2 | 0 | 1 | / | 1 | 2 | 1 | 1 | 22 | 1.294118 |

/: the missing tooth
